# Thermal-plex: fluidic-free, rapid sequential multiplexed imaging with DNA-encoded thermal channels

**DOI:** 10.1038/s41592-023-02115-3

**Published:** 2023-12-27

**Authors:** Fan Hong, Jocelyn Y. Kishi, Ryan N. Delgado, Jiyoun Jeong, Sinem K. Saka, Hanquan Su, Constance L. Cepko, Peng Yin

**Affiliations:** 1 Wyss Institute for Biologically Inspired Engineering, Harvard University, Boston, MA USA; 2 Department of Systems Biology, Harvard Medical School, Boston, MA USA; 3 Departments of Genetics and Ophthalmology, Blavatnik Institute, Harvard Medical School, Boston, MA USA; 4 Howard Hughes Medical Institute, Chevy Chase, MD USA; 5 Present Address: Department of Chemistry, University of Florida, Gainesville, FL USA; 6 Present Address: Genome Biology Unit, European Molecular Biology Laboratory (EMBL), Heidelberg, Germany

**Keywords:** Fluorescence imaging, Biotechnology

## Abstract

Multiplexed fluorescence imaging is typically limited to three- to five-plex on standard setups. Sequential imaging methods based on iterative labeling and imaging enable practical higher multiplexing, but generally require a complex fluidic setup with several rounds of slow buffer exchange (tens of minutes to an hour for each exchange step). We report the thermal-plex method, which removes complex and slow buffer exchange steps and provides fluidic-free, rapid sequential imaging. Thermal-plex uses simple DNA probes that are engineered to fluoresce sequentially when, and only when, activated with transient exposure to heating spikes at designated temperatures (thermal channels). Channel switching is fast (<30 s) and is achieved with a commercially available and affordable on-scope heating device. We demonstrate 15-plex RNA imaging (five thermal × three fluorescence channels) in fixed cells and retina tissues in less than 4 min, without using buffer exchange or fluidics. Thermal-plex introduces a new labeling method for efficient sequential multiplexed imaging.

## Abstract (web summary)

The thermal-plex method for highly multiplexed imaging uses DNA probes activated when briefly elevated to designated temperatures for rapid, fluidics-free sequential imaging in cells and tissues.

## Main

Multiplexed imaging of cell and tissue samples with methods such as fluorescence in situ hybridization (FISH) for nucleic acid targets and immunofluorescence (IF) for protein targets can reveal critical details about the abundance and spatial organization of molecular targets^1,2^. However, standard microscope setups only allow three to five distinct fluorescent dyes to be identified due to overlapping emission spectra, limiting the number of targets that can be visualized simultaneously. This limitation can be overcome by sequential imaging methods that rely on iterative rounds of target labeling and imaging^3–6^. More recently, DNA-based barcoding and exchange methods, which stain all targets with DNA-barcoded affinity reagents (FISH probes or antibodies) in one round, followed by iterative rounds of fluorescently labeled oligonucleotide (imager) binding and washing, have been successful at imaging large numbers of DNA, RNA and protein targets that have DNA-based barcodes attached^7–14^. When paired with combinatorial readout, the number of distinguishable targets can be increased exponentially in cases where they are spatially separated^15–21^.

Although buffer-exchange-based methods allow higher multiplexing, they involve complex setup, laborious operation and long signal channel transition times. Samples may need to be removed from the microscope for buffer exchange, which requires relocating the region(s) of interest when the sample is placed back on the stage. The sample may also become damaged if a coverslip needs to be removed at each step. If buffer exchange is performed on a stage manually, there is a constant risk of losing focus as well as disturbing and contaminating the sample. Custom fluidics apparatuses are available for automated fluidics^22^, but can be expensive and require frequent monitoring to avoid air bubbles and failures in tubing connections, which may be a barrier to routine use across laboratories. Finally, buffer exchange is typically followed by long incubations (tens of minutes to an hour) for labeling and de-labeling, which can substantially increase the on-scope instrument time and limit speed and throughput.

**Fig. 1:**
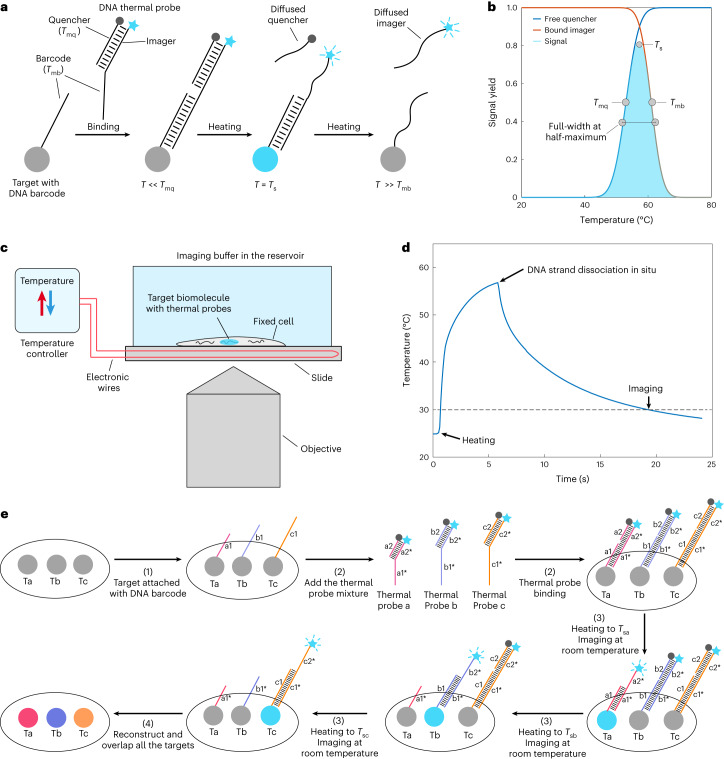
Schematic of thermal-plex imaging. **a**, The thermal-plex imaging concept is based on stepwise melting of DNA thermal probes from an in situ target. After labeling the target biomolecule with a DNA barcode, the thermal probe set comprising a quencher strand and an imager strand is hybridized to the DNA barcode. At temperatures lower than the melting temperature of the quencher, the fluorescent signal is quenched. When the temperature is heated to the signal temperature, the quencher is melted off and fluorescence signal is emitted and can be imaged, including after cooling the sample to room temperature. Signal is then removed by heating to a temperature substantially higher than the melting temperature of the barcode domain (to which the imager binds). **b**, Thermal spectrum of an exemplary thermal-plex probe set. *T*_s_ is the temperature that gives maximum fluorescent signal. *T*_mq_ is the melting temperature of the quencher. *T*_mb_ is the melting temperature of the barcode domain. The width is the distance between the half maximum of the signal. **c**, Imaging setup for thermal-plex. Cells are seeded on a slide with an on-scope temperature control module, which can change the slide temperature rapidly in seconds to dissociate DNA strand into the imaging buffer. **d**, Temperature profile of the slide during the heating and cooling process. It takes about 5 s to reach the desired signal temperature (57 °C) to melt off the DNA strands. After the temperature is cooled down to around 30 °C, the sample is imaged. **e**, Example process of multiplexed fluorescent imaging for three targets (Ta, Tb and Tc) with thermal-plex. (1) Single-stranded DNA barcodes (a1, b1 and c1) are attached to different target molecules. (2) Orthogonal DNA thermal probe sets (a, b and c) are hybridized to the DNA barcodes. (3) The target Ta is visualized at room temperature after the heating to signal temperature *T*_sa_ to activate the fluorescence signal. Several other targets are visualized sequentially after heating to their assigned signal temperatures (*T*_sb_, *T*_sc_). (4) The images are aligned computationally and reconstructed to overlay all the targets.

Here we describe the concept of buffer-exchange-free thermal multiplexed fluorescence imaging (thermal-plex), which overcomes these limitations by engineering programmable melting of DNA probes in situ with an on-scope temperature controller that rapidly changes temperature to create thermal imaging channels (Fig. 1a). Compared with existing methods, thermal multiplexing provides greater accessibility and is compatible with conventional microscope setups without sensitive detector calibration or complex instrumentation. The switching time between thermal channels in thermal-plex can be completed within 30 s, which is much faster than the transition time in the buffer-exchange-based method (generally ~30 mins to hours^15,16^). Furthermore, as the sample is heated directly on the microscope for a relatively short time (seconds), it remains well preserved, and the field of view and focus can be maintained easily throughout, eliminating extra adjustment steps. We show how the thermal channels that we engineer can be used for multiplexed imaging in fixed cells and tissue samples, and how they can be combined with spectral channels (five thermal × three spectral) for 15-channel imaging of RNA in less than 4 min, without requiring buffer exchange or fluidics.

## Results

### Principle of the DNA thermal-plex method

**Extended Data Fig. 1:**
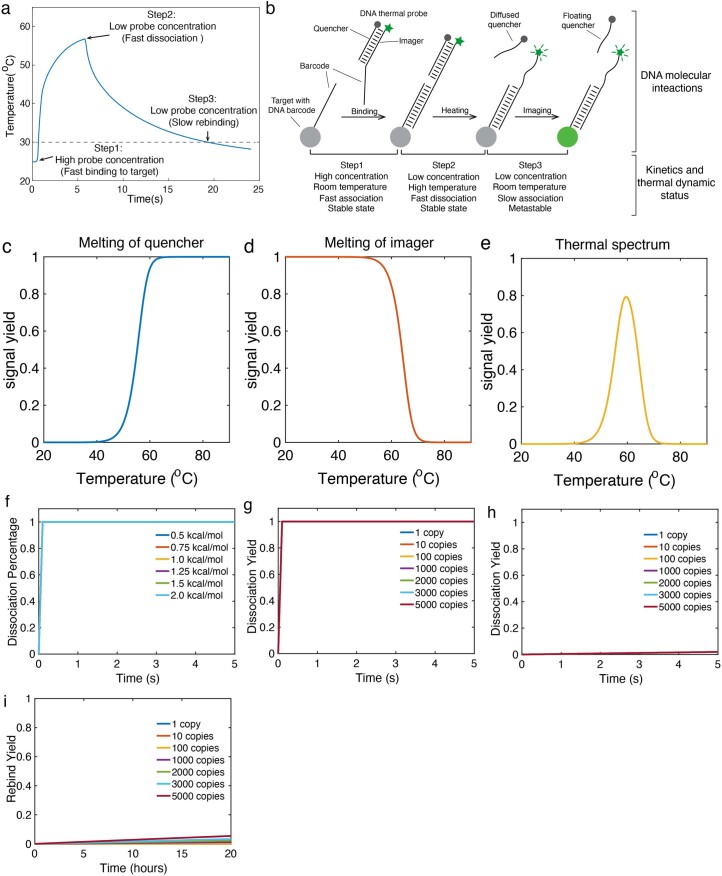
The mechanism and simulation of DNA probe binding for the thermal-plex imaging. **a**, The schematic illustration of three steps. **b**, The schematic illustration of DNA imager and quencher interactions and their kinetic and thermal dynamic status in these three steps. **c**, Plot showing the relationship between the signal yield and the temperature in the quencher melting step. **d**, Plot showing the relationship between the signal yield and the temperature in the imager melting step. **e**, the thermal spectrum of a DNA thermal-plex probe set is calculated as the multiplication of the imager and quencher yields. **f**. Kinetics of strand dissociation under different binding energy *ΔG* with RNA copy number of 1000. **g**. Kinetics of strand dissociation *in situ* is predicted to take less than 1 second at the signal temperature (57 °C). **h**. Kinetics of strand dissociation *in situ* is minimal at lower temperature channels (48 °C), **i**. After the sample is cooled to around 30 °C for imaging, rebinding of the strand is predicted to be extremely slow.

The core part of the thermal-plex method is the design of thermal DNA probes that allow efficient fluorescent signal generation within programmed temperature ranges (thermal channels) and but not in other orthogonal thermal channels. The DNA thermal probe is composed of a DNA imager strand and a quencher strand, which are prehybridized to quench the fluorescent signal. In our design, affinity reagents (such as FISH probes or DNA-conjugated antibodies) contain single-stranded DNA barcode overhangs (barcode strands). Each barcode is associated with the imager strand in a thermal probe. The fluorescent imager comprises two regions: (1) a strong binding region complementary to the DNA barcode on the affinity reagent, where the melting temperature is *T*_mb_; (2) a weaker binding region for a complementary quencher strand, where the melting temperature is *T*_mq_. (Fig. 1a). To implement the DNA thermal probe for imaging, there are three main steps: (1) binding of DNA thermal probes to the DNA barcodes on the affinity reagent; (2) melting of the quenchers at elevated temperature to activate fluorescent signal; (3) imaging of the targets with standard fluorescence microscope setup (Supplementary Note 1 and Extended Data Fig. 1).

In the first step, at temperatures substantially (generally 3–5 °C) lower than *T*_mq_, when the quencher strand is bound to the imager strand, and the imager strand is bound to the DNA barcode, the fluorescence signal is quenched. In the second step, within a specific intermediate temperature range (substantially higher than *T*_mq_ but lower than *T*_mb_), the quencher strand dissociates from the barcode, but the imager remains stably bound to the barcode strand and emits fluorescence signal. The dissociation of the quencher is rapid (within seconds) in this step because of elevated temperature and low probe concentration in the imaging solution. In this step, through thermodynamic simulation of melting profiles, we modeled the binding properties of the strands and predict the temperature range in which imagers will be bound to the barcode strands and fluoresce (Supplementary Note 2 and Extended Data Fig. 1). The example thermal profiles of a quencher and an imager are shown in Fig. 1b. The thermal profile intersection of quencher strand dissociation and imager strand dissociation region defines the thermal range in which fluorescence will be seen at a specific target site. The temperature with the highest fluorescent signal is defined as the signal temperature *T*_s_ for this DNA thermal probe. The full-width at half-maxima (*W*) of the thermal spectra determine how many thermal channels can be achieved across the temperature range from room temperature to 75 °C. In a typical thermal-plex imaging process, the sample is incubated on a slide with an on-scope temperature control module (Fig. 1c). A heating spike is applied to rapidly change the temperature to the signal temperature for DNA strand dissociation (Fig. 1d). After probes have dissociated from their targets, they will diffuse out of the microscope focal plane.

In the third step, imaging is be performed after the removal of the quencher by melting. The melted quencher and imager will stay in a diffused metastable state in the imaging solution because of low concentration (fM–pM). The rate of rebinding to the targets is extremely slow and the signal caused by the diffused probe in the background is negligible (see Supplementary Note 3 and Extended Data Fig. 1 for detailed analysis). Therefore, the fluorescent signal intensity for the target is dependent primarily on the equilibrium in the second step as shown in the thermal spectra (Fig. 1b). In this step, samples can be cooled down for imaging at room temperature to avoid the undesirable effects on the imaging of elevated temperature.

Then, the next cycle of thermal-plex imaging starts over again in subsequent thermal channels. The temperature in the next channel is substantially higher than *T*_mb_; the imager strand dissociates from the barcode strand and fluorescence signal at this site is removed without interfering with the signal in the next channel. As the dissociated imager and quencher stay in a diffused metastable state in the third step, we can model the melting profile of DNA thermal probes in the second step to predict the fluorescent signal in the third imaging step. The programmability and predictability of basepairing allows the robust design of several separate thermal channels. By modulating the sequence composition and length of imager and quencher binding domains, we can design several orthogonal DNA thermal probes sets that emit fluorescence in different temperature ranges (Fig. 1e). By then coupling orthogonal thermal probe sets to in situ hybridization (ISH) probes or other affinity reagents, fluorescence signals can be generated sequentially for several biological targets by successively applying heating spikes at several signal temperatures. It should be noted that the temperature-induced melting and resultant signal removal from the target is irreversible, and the target cannot be imaged again unless the target barcodes are relabeled with the corresponding DNA thermal probe, similar to other sequential imaging methods^7,9^.

### Simulation guided design of thermal channels with DNA thermal probes for multiplexed imaging

**Extended Data Fig. 2:**
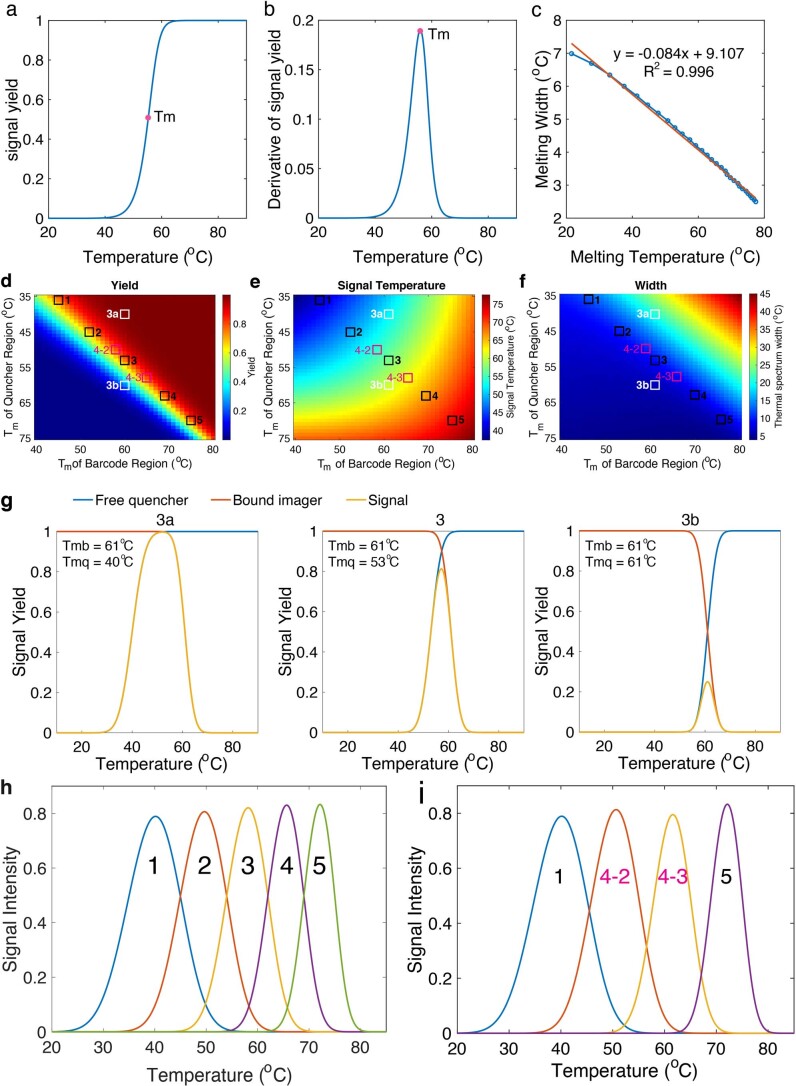
The thermal spectral of DNA thermal-plex imaging. **a**. A typical melting curve with melting temperature of 57 °C. **b**. The derivative of the melting curve is fitted with the Gaussian function. **c**. The correlation between the width of melting curve and melting temperature. The signal yield (**d**), signal temperature (**e**), and width of the thermal spectra (**f**) with different combinations of quencher and DNA barcode melting temperature. See Supplementary Note 3 for detailed discussion. The selected representative melting profiles are shown in squares. **g**, Examples of three representative thermal spectra with different combinations of the quencher and binding domain melting temperatures. Examples 3a and 3b are suboptimal thermal spectra due to high spectrum width and low signal yield, respectively. The spectrum in 3 is a more optimal design with relatively higher signal yield and narrower width. **h**, The thermal spectra of five near optimal thermal channels (39 °C, 48 °C, 57 °C, 65 °C, 72 °C) with minimal crosstalk and relatively high signal yield. **i**, The thermal spectra of the four stringent combinations. The signal temperatures were determined to be 40 °C, 52 °C, 62 °C, and 72 °C.

To implement multiplexed DNA thermal-plex for cellular imaging, each set of DNA thermal probes needs to have (1) sufficient fluorescent signal generated when heated to its signal temperature; (2) minimal fluorescence signal when heated to signal temperatures for other orthogonal probe sets and (3) a large number of nonoverlapping thermal channels to increase multiplexity. To create maximum set probe sets with nonoverlapping thermal spectra, we explored the design space systematically with computational simulations under all combinations of melting temperatures of barcode regions (from 40 °C to 80 °C) and quencher regions (35 °C to 75 °C, see Supplementary Note 4, Extended Data Fig. 2). The resultant heatmaps of the predicted signal yield, the signal temperature, and the width of the thermal spectra are shown in Extended Data Fig. 2d–f.

Probe sets residing in the upper-right region of the heatmap have higher signal yield but wider thermal spectra (Extended Data Fig. 2g), and probe sets residing in the lower-left triangular area have lower signal yield but narrower thermal spectra. The optimal region is therefore in the diagonal area of the heatmap, corresponding to the probes that give relatively high signal yield (>0.8) while still maintaining relatively narrow thermal spectra. The details of three representative cases (shown with 3 black, 3a and 3b white squares in the heatmap) located in those three areas are shown in Extended Data Fig. 2d–f. Other than the relatively high yield, the selected thermal spectra need to have minimal crosstalk (signal overlap is set to be <0.12 in this study) with each other to produce unambiguous signal and allow a sufficiently large number of thermal channels. We opted to set the number of thermal channels to five in this study. A more stringent panel with four thermal channels can also be designed as shown in Extended Data Fig. 2. Furthermore, during the sample preparation and labeling step before the imaging, the excess unbound probes are washed away from the fixed cells or tissues to reduce the fluorescent background. Therefore, the lowest signal temperature of the thermal-plex imaging needs to be higher than the washing condition (usually 30–37 °C in the experiments in this study) to ensure high yield of the signal in the lowest temperature thermal channel. Based on the above criteria (for example, signal yield, signal crosstalk, lowest temperature thermal channel), five optimal combinations (indicated as one, two, three, four and five black squares) were selected to create five thermal channels with minimal signal crosstalk, as shown Extended Data Fig. 2h. The signal temperatures were designed to be 39 °C, 48 °C, 57 °C, 65 °C and 72 °C, respectively.

### Validation of in situ RNA imaging with DNA thermal probes

To validate the method, a DNA thermal probe set was designed to target the *APC* mRNA transcript in HeLa cells. This ubiquitously expressed gene is involved in several cellular processes including tumor repression, cell division and cell organization in a tissue^23^. The melting temperatures of the quencher and barcode regions were designed to be 53 °C and 61 °C, respectively. The resulting optimal signal temperature was predicted to be approximately 57 °C. We first designed ISH probes for *APC* using Oligominer^24^. Thermal probe barcode regions were then appended to the 3′ end of each ISH probe sequence. We also added reference probe binding regions to the 5′ end of each ISH probe sequence where a reference imager strand would be stably bound and fluorescent across all temperatures as a positive control. Appended reference and thermal probe sequences were screened against the human transcriptome using Blast to reduce the risk of nonspecific binding and background signal^25^.

**Fig. 2:**
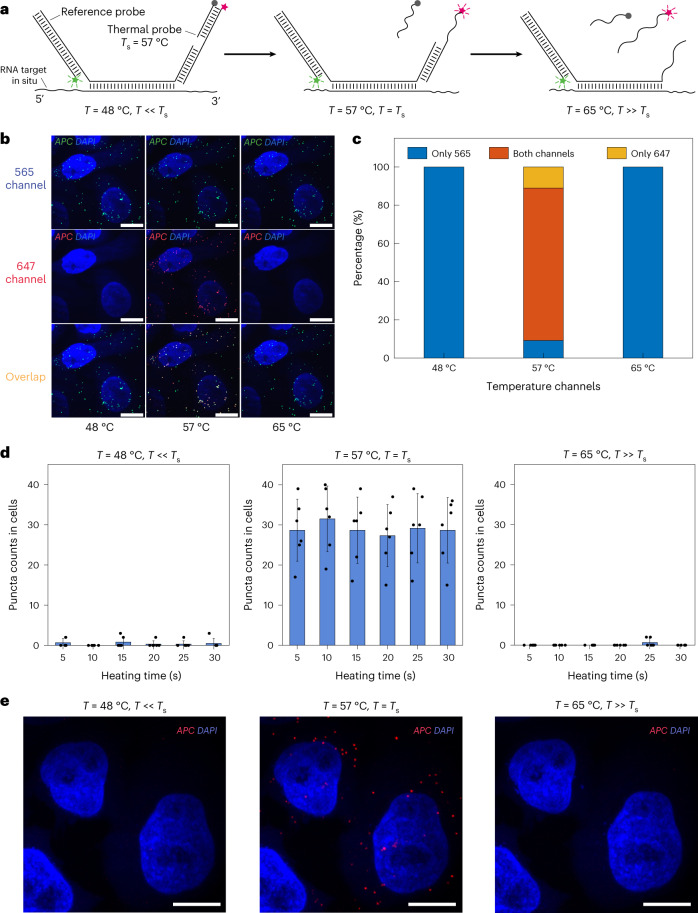
Validation and fast channel switching speed of DNA thermal-plex for RNA FISH in situ. **a**, Design for the reference and thermal probe sets. Reference probes fluoresce at all temperatures, while the thermal probe set fluoresce only after being exposed to a heating spike at its signal temperature (for example, 57 °C). **b**, FISH imaging of *APC* RNA transcripts in fixed HeLa cells with a 57 °C thermal probe set in 565-nm channel, 647-nm channel and with both channels overlaid, after transiently exposed to heating spikes at three different temperatures. Only after being heated to the signal temperature 57 °C did the thermal probe show the fluorescence signal, which appear to be colocalized with the reference probe. **c**, Colocalization analysis of the puncta in 565-nm and 647 channels, showing percentages of the puncta that are detected only in 565-nm channel (blue), only in 647-nm channel (red) and colocalized in both channels (orange). A total of four cells were analyzed. **d**, Barplots of puncta counts in cells imaged after heating for different amounts of time below (48 °C), at (57 °C) and above (65 °C) the signal temperature. Error bars depict s.d. between cells. **e**, FISH images of *APC* gene after heating at different temperatures (48 °C, 57 °C and 65 °C) for 5 s. A total of six cells were analyzed for **d**. Three independent experiments were repeated for validation. Scale bars, 10 µm. Source data

**Extended Data Fig. 3:**
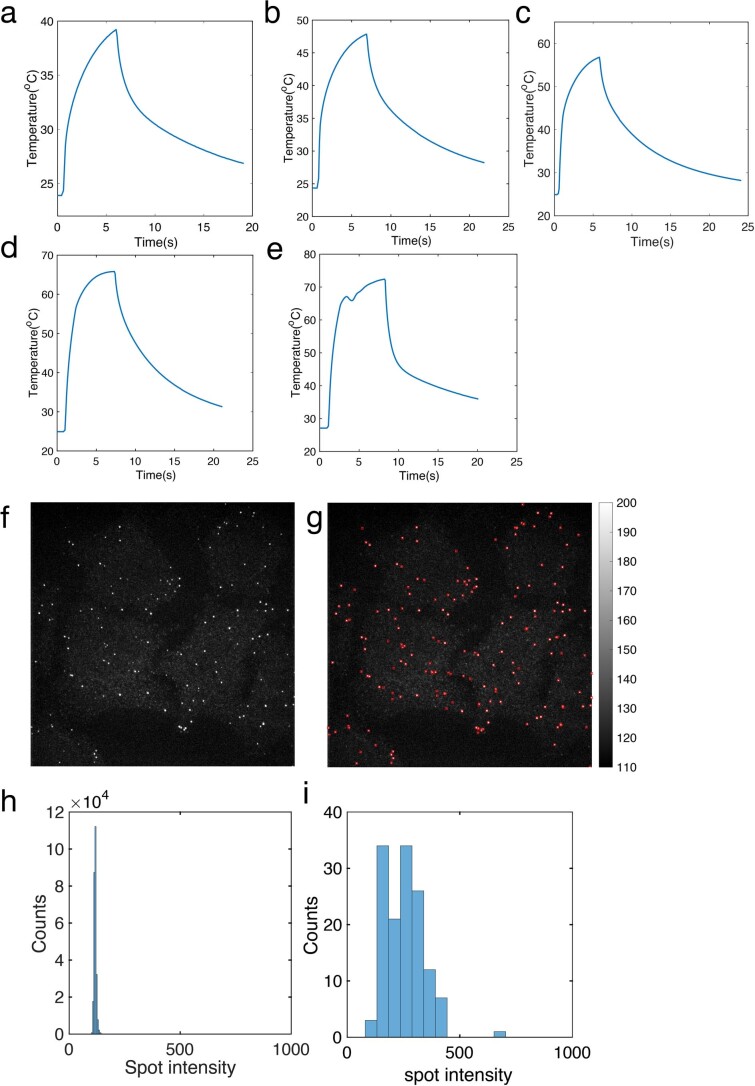
The temperature control and RNA image analysis. **a**. Heating to signal temperature at 39 °C. **b**. Heating to signal temperature at 48 °C. **c**. Heating to signal temperature at 57 °C, **d**. Heating to signal temperature 65 °C, **e**. Heating to signal temperature 72 °C. The device rapidly heats to the desired temperature and melts off the quencher and imager strand *in situ*. **f**, Exemplary image of RNA FISH from DNA thermal-plex. **g**, RNA puncta that were identified by an intensity filter are indicated with red boxes. **h**, The histogram of all pixels in the field of view. **i**, Histogram of identified RNA spots.

To test the thermal probes for RNA imaging in situ, HeLa cells were cultured and fixed on a substrate with a temperature control module. Following our standard overnight in situ hybridization protocol^12^, we bound the reference imager and the thermal probes to the barcode overhangs on the ISH probes (Fig. 2a). Thermal probes were preassembled before in situ binding to the barcodes to reduce the background introduced by the imager strands. The reference imagers were labeled with Atto565 dye, and the thermal probes were labeled with Alexa647 dye. Iterative heating spikes and room temperature imaging steps were then performed to profile fluorescence signal across temperatures (Extended Data Fig. 3). We confirmed that, after temperature spikes lower than the signal temperature, such as 48 °C, only the 565-nm channel with reference probes showed the RNA puncta signal (Fig. 2b). The nonobservable fluorescent signal in the 647-nm channel indicated good quenching efficiency for the thermal probes at this lower temperature. After heating to the signal temperature (57 °C) and cooling down to the image acquisition temperature (~30 °C), both the 565-nm and 647-nm channels showed fluorescent and overlapping RNA puncta signal, confirming temperature-dependent thermal probe fluorescence. Finally, after the application of a higher temperature spike of 65 °C, only the 565-nm channel showed puncta signal, indicating that the imager strand had dissociated successfully from the ISH probes.

**Extended Data Fig. 4:**
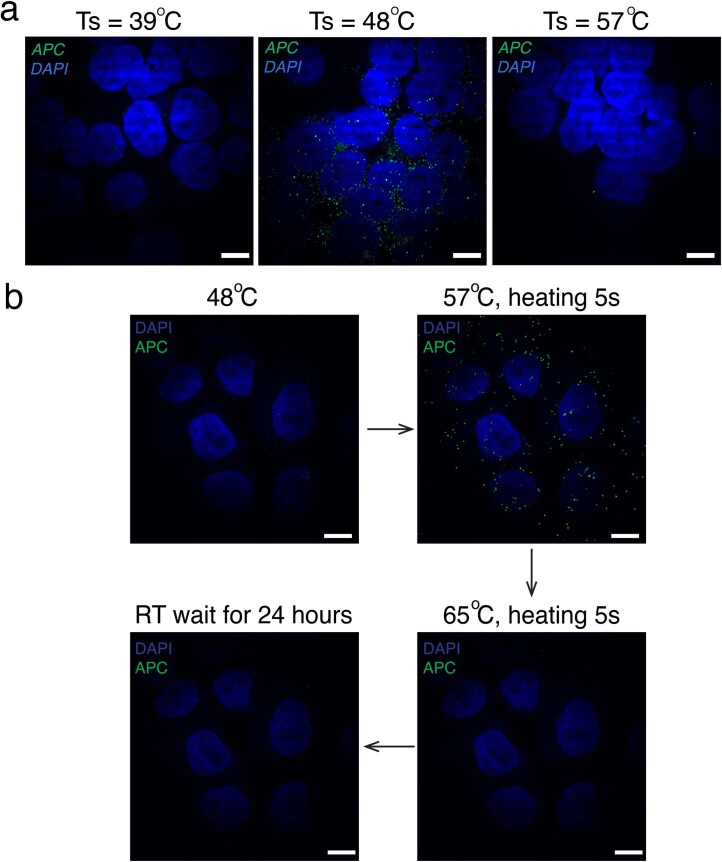
The universality for different cell lines and robustness of probe dissociation of thermal-plex imaging. **a**, Implementation of thermal-plex in the HEK293 cell line. The APC ISH probe is designed to bind DNA thermal probes with signal temperature at 48 °C. Only when the temperature is heated to 48 °C, the RNA puncta signal will show up. The scale bars are 10 µm. b. The negligible rebinding of imager in thermal-plex during thermal channel switching. DNA thermal probe with signal temperature at 57 °C is implemented to visualize APC mRNA in Hela cells. The signal will only show up when a 57 °C heating spike is applied. When switching to next temperature channel, the imager dissociates and is not observed to rebind even after 24 hours. All the scale bars are 10 µm. At least three independent repeats were performed to validate the results.

The puncta information was analyzed for different temperature and fluorophore channels as shown in Fig. 2c using custom MATLAB code (Extended Data Fig. 3). We found that 88.5% of the puncta in the 565-nm channel and 86.3% of the puncta in the 647-nm channel were well aligned at 57 °C. The high correlation of signal across the reference probes and thermal probes demonstrates that thermal probes can illuminate target RNA under designed signal temperatures, with the signal diminished successfully at lower or higher temperatures. The method was also validated with the HEK293 cell line (Extended Data Fig. 4).

### Fast dissociation and slow reassociation kinetics for thermal channel switching and imaging

As signal in thermal channels relies only on the dissociation of the quencher and imager strand at the signal temperature, the speed of fluorescence generation is predicted to be very fast (within seconds) based on kinetic simulations (Supplementary Note 2). To characterize the temperature channel switching time, fluorescence imaging data were collected after heating the substrate to a temperature substantially lower than the signal temperature, at the signal temperature, and substantially higher than the signal temperature for different amounts of time (Fig. 2d,e). We found that 5 s (heating from room temperature to signal temperature) was enough to fully melt off the thermal quencher strand and generate the signal at the signal temperature and remove thermal imager strands above the signal temperatures. As the dissociation of the quencher and imager strands is almost instantaneous at signal temperature, the speed of channel switching is limited by the speed of the temperature control of the on-scope heating device and the time for subsequent cooling to imaging acquisition temperature.

Although the heat-induced quencher strand and imager strand dissociation is fast, rebinding of these dissociated strands is extremely slow as the concentration of diffused imager strands is low (femtomolar–picomolar range), allowing us to cool down the sample and take images at ~30 °C with negligible background fluorescence (see Supplementary Note 2 for detailed analysis). We observed negligible rebound signal, with the thermal channel switching to a higher one after 24 h (Extended Data Fig. 4).

### Validation of five thermal channels for fluorescence imaging

**Fig. 3:**
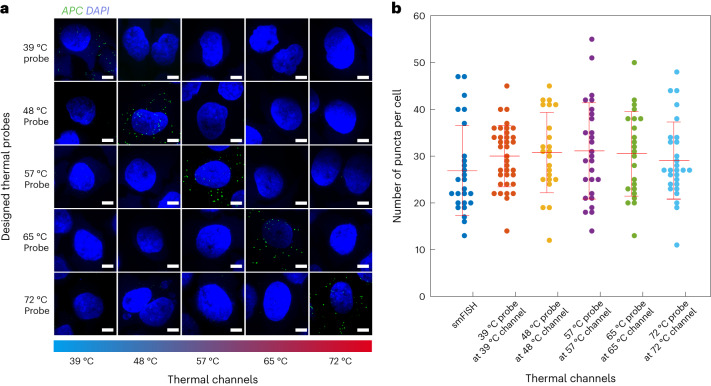
Validation of five thermal channels with minimal signal crosstalk. **a**, FISH images for five distinct thermal channels at different temperatures (39 °C, 48 °C, 57 °C, 65 °C and 72 °C) all targeting the *APC* mRNA. All probes show only strong RNA puncta signal in their designated temperature channel. Scale bars, 5 µm. **b**, Plot of thermal-plex puncta for all the five thermal probes in their corresponding thermal channels. smFISH puncta are used as the positive control. All five thermal channels yielded similar quantitative *APC* expression in HeLa cells. The numbers of cells used to count the fluorescent puncta for smFISH and all five thermal channels were 26, 36, 23, 27, 23 and 28, respectively. The red line shows the mean expression level of *APC* mRNA. Data are presented as mean values ± s.d. Source data

After validating the thermal-plex mechanism, we then sought to validate the five designed thermal channels (39 °C, 48 °C, 57 °C, 65 °C, 72 °C) designed to have minimal signal crosstalk and described in Extended Data Fig. 2h (see Supplementary Table 1 for probe sequence). Each of the five channels were validated with the *APC* probe set design described in Fig. 3, and crosstalk between channels was profiled by analyzing the number of RNA puncta per cell for each designed thermal probes at their respective signal temperatures (Fig. 3a). All five thermal DNA probe sets showed expected fluorescent signal level at their prescribed signal temperatures and minimal signal at signal temperatures for orthogonal probe sets. We further analyzed the number of RNA puncta per cell for each thermal probe set and found these counts to be consistent across all thermal channels (Fig. 3b).

### Multiplexed cellular RNA imaging

**Fig. 4:**
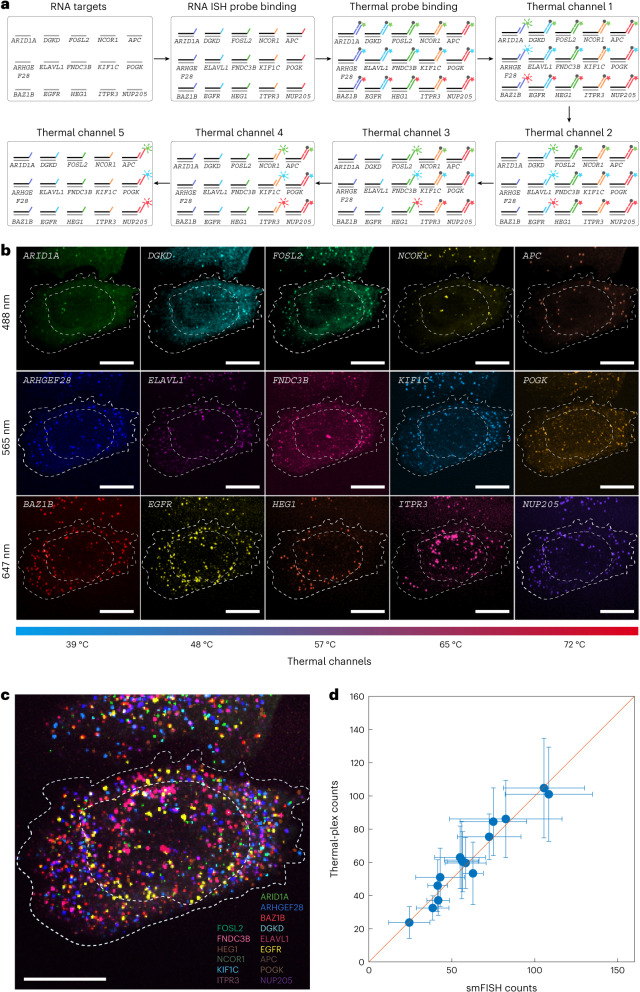
Fifteen-plex RNA imaging with the combination of five thermal channels and three fluorescence channels in fixed cells. **a**, Schematic for 15-plex RNA imaging. Fifteen ISH probe sets with distinct DNA barcodes are hybridized in situ. Fifteen orthogonal DNA thermal probe sets are then hybridized to ISH probe DNA barcodes. Following each iterative round of heating to signal temperatures, fluorescence images in three channels are collected after the sample is cooled to ~30 °C. **b**, Individual channel images for each of the 15 RNA targets. **c**, Overlaid images for all 15 mRNAs in a single cell resolved by thermal-plex. See Extended Data Fig. 9 for the computationally reconstructed image. **d**, Comparison of the resolved 15-plex single-cell RNA expression between thermal-plex and conventional smFISH methods. The red line indicates *y* = *x*. The high linear correlation (*R*^2^ = 0.95) between the two methods indicates the robustness of the thermal-plex imaging methods. The s.d. shows that the total cell numbers for the thermal-plex and smFISH are 32 and 38 cells, respectively. Data are presented as mean values ± s.d. Scale bars, 10 µm. Source data

**Extended Data Fig. 5:**
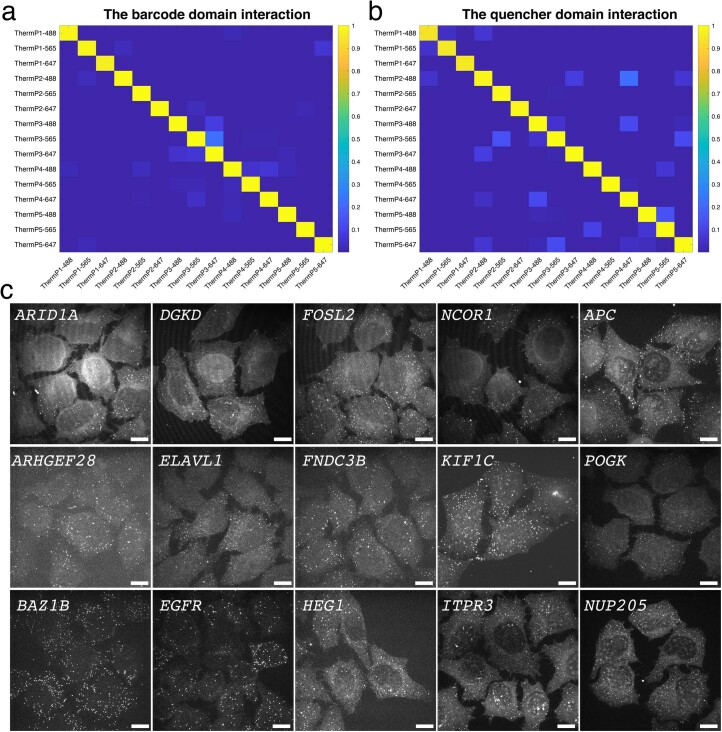
The orthogonality of designed 15 thermal probes and smFISH images of 15 RNA targets. **a**, Orthogonality matrix of the barcode domains for 15 designed thermal probes. **b**, Orthogonality matrix of the quencher regions for the 15 designed thermal probes. The designed sequence is orthogonal to each other and none of them has strong interactions predicted to affect the imaging. The sequence interactions were evaluated with NUPACK software to calculate the yield at equilibrium. **c**, The validation of 15 RNA targets with smFISH in Hela cells. Each RNA was bound individually with ISH probes and then the imager was applied to visualize the RNA in cells. Scale bars, 10 µm. At least three independent repeats were performed to validate the results.

**Extended Data Fig. 6:**
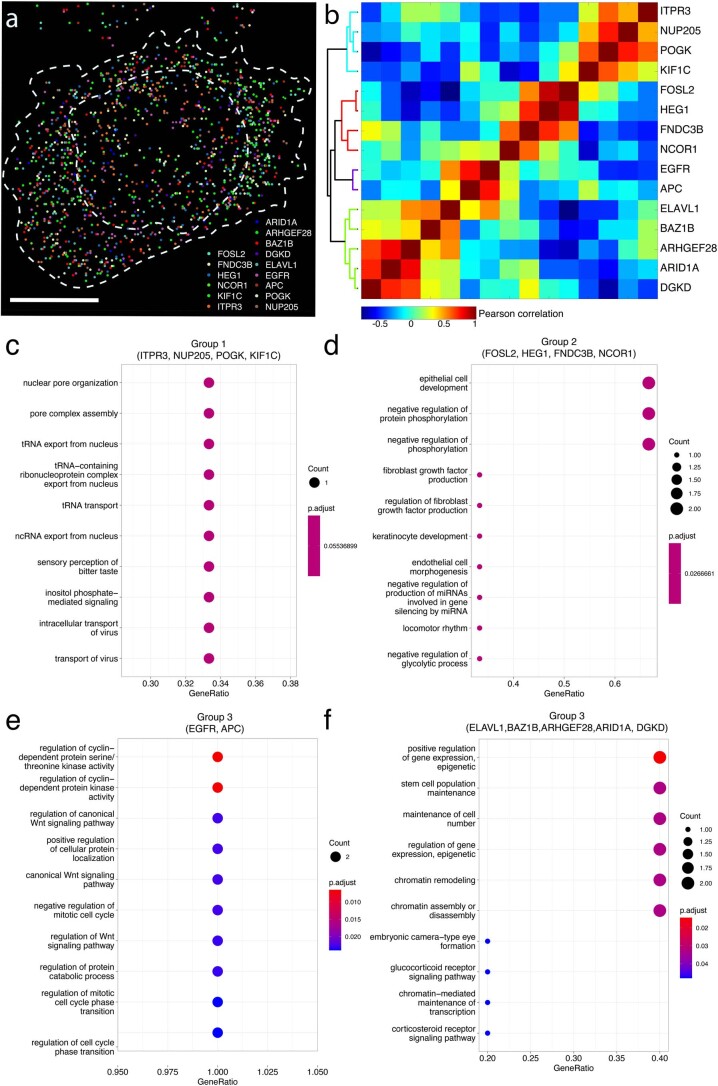
Signal crosstalk validation of 5 thermal probes for 488 nm channel. **a**, RNA FISH images for ARID1A targeting with Atto488-labeled imagers for the 39 °C thermal channel and RNA copies per cell for all fluorescent and thermal channels. There is no notable signal crosstalk between the different thermal channels. **b**, Validation for the thermal probe set targeting the DGKD transcript with Atto488-labeled imagers for the 48 °C thermal channel. **c**, Validation for FOSL2 transcript thermal probe set with Atto488-labeled imagers for the 57 °C thermal channel. **d**, Validation for NCOR1 transcript thermal probe set with Atto488-labeled imagers for the 65 °C thermal channel. **e**, Validation for APC transcript thermal probe set with Atto488-labeled imagers for the 72 °C thermal channel.

**Extended Data Fig. 7:**
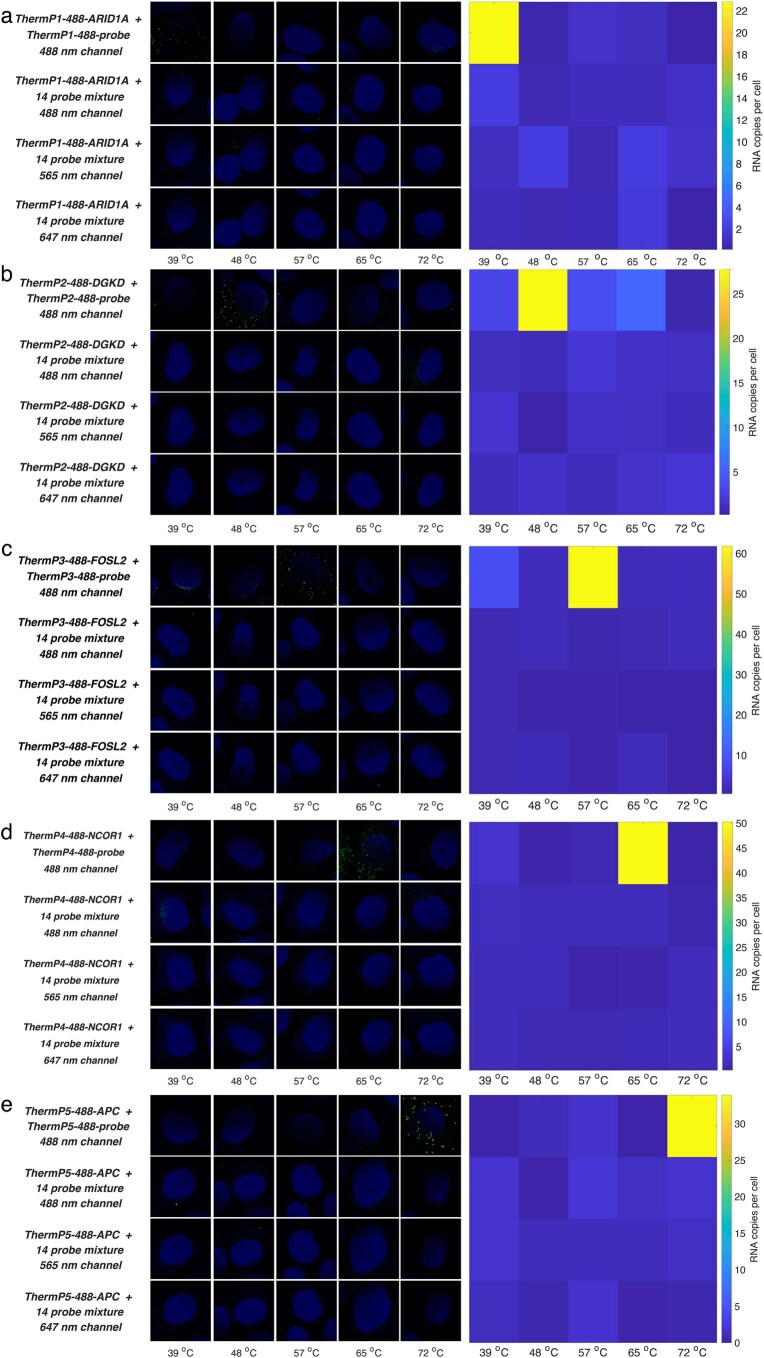
Signal crosstalk validation of 5 thermal probes for 565 nm channel. **a**, RNA FISH images for BAZ1B target with Atto565-labeled imagers for the 39 °C thermal channel and RNA copies per cell for all fluorescent and thermal channels. There is no notable signal crosstalk between the different thermal channels. **b**, Validation for the thermal probe set targeting the EGFP transcript with Atto488-labeled imagers for the 48 °C thermal channel. **c**, Validation for HEG1 transcript thermal probe set with Atto488-labeled imagers for the 57 °C thermal channel. **d**, Validation for POGK transcript thermal probe set with Atto488-labeled imagers for the 65 °C thermal channel. **e**, Validation for NUP205 transcript thermal probe set with Atto488-labeled imagers for the 72 °C thermal channel.

**Extended Data Fig. 8:**
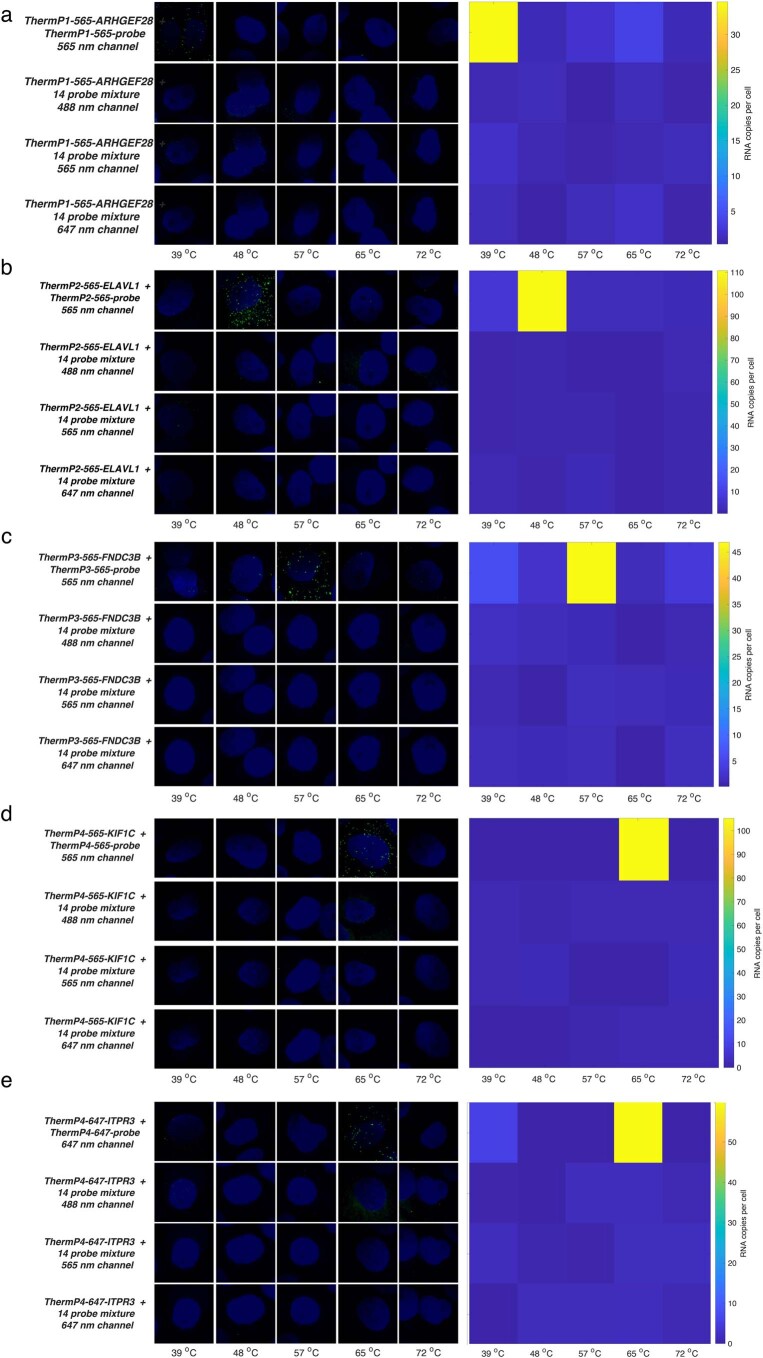
Signal crosstalk validation of 5 thermal probes for 647 nm channel. **a**, RNA FISH images for *ARHGEF28* target with Alexa647-labeled imagers for the 39 °C thermal channel and RNA copies per cell for all fluorescent and thermal channels. There is no notable signal crosstalk between the different thermal channels. **b**, Validation for the thermal probe set targeting the *ELAVL1* transcript with Alexa647-labeled imagers for the 48 °C thermal channel. **c**, Validation for *FNDC3B* transcript thermal probe set with Alexa647-labeled imagers for the 57 °C thermal channel. **d**, Validation for *KIF1C* transcript thermal probe set with Alexa647-labeled imagers for the 65 °C thermal channel. **e**, Validation for *ITPR3* transcript thermal probe set with Alexa647-labeled imagers for the 72 °C thermal channel.

The thermal-plex method provides an additional dimension for multiplexing on top of spectral multiplexing achievable with distinct fluorescence channels. A wide range of fluorophore and quencher pairs^26^ can be used to implement the DNA thermal-plex for higher-plex imaging. To show how thermal and spectral multiplexing can be combined to substantially increase multiplexing capability (to 15-color) without requiring buffer exchange, we combined each of our five thermal channel sets with three different fluorescence channels (Atto488, Atto565 and Alexa647) to visualize 15 biological targets simultaneously. As the fluorophore and quencher located at the DNA thermal probe duplex are positioned sufficiently close to effect contact quenching, the quenching efficiency is expected to be over 95%^26^. We designed 15 sets of thermal probes to target 15 different mRNA transcripts in HeLa cells (see Supplementary Table 2 for sequence information). These RNAs have diverse functions in the cell, including gene expression regulation (*ARID1A, BAZ1B, FOSL2, NCOR1, POGK, ELAVL1*), cellular transportation (*NUP205*, *KIF1C*), cell adhesion (*FNDC3B*), cellular pathway regulation (*DGKD*, *HEG1*, *ITPR3*, *APC*), cell growth regulation (*EGFR*) and cellular aggregation (*ARHGEF28*) (see Supplementary Table 3 for primary probe sequence information). Each set of the three thermal probes designed to have the same signal temperature was encoded with three different fluorophores, Atto488, Atto565 and Alexa647, respectively (Fig. 4a). The 15 thermal probes were designed to be orthogonal with an in silico hybridization test (Extended Data Fig. 5). Gene expression variance between the cells is dependent on the cell cycle state. We further validated the RNA targets with the conventional smFISH method (Extended Data Fig. 5). To test probe orthogonality experimentally, we applied each designed barcode to bind 14 noncognate thermal probes in situ to test the signal crosstalk (see Extended Data Fig. 6 for thermal probes in 488-nm channel, Extended Data Fig. 7 for thermal probes in 565-nm channel and Extended Data Fig. 8 for thermal probes in 647-nm channel), and observed only negligible crosstalk signal.

**Extended Data Fig. 9:**
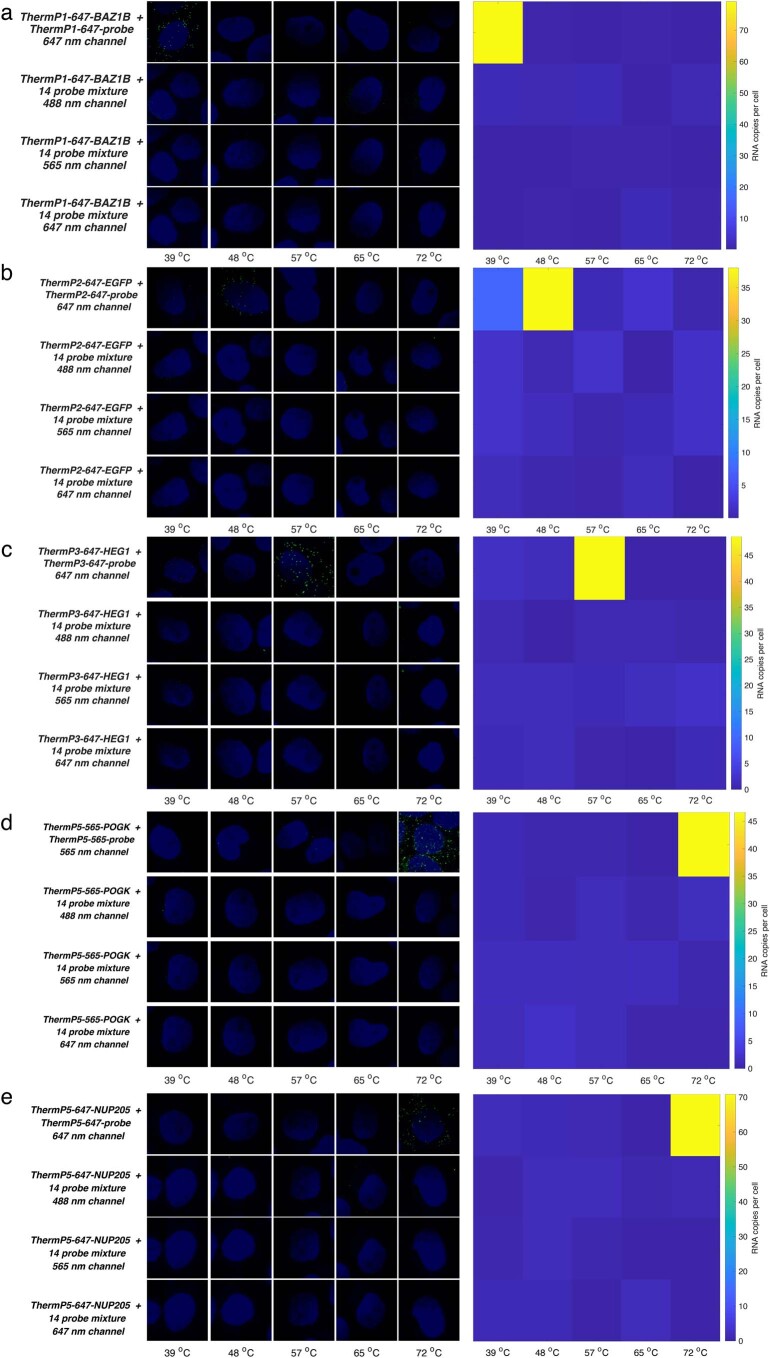
Single cell analysis of the resolved 15 mRNAs. The reconstructed spatial distribution of the 15 mRNAs. The puncta position information was extracted from the fluorescent images of 15 channels and then reconstructed to overlay all 15 mRNAs. The cell and nucleus boundaries were shown in dashed lines. The scale bar is 10 µm. **b**, Matrix of pairwise correlation coefficients of cell-to-cell variation in gene expression for the measured 15 mRNAs and the hierarchical clustering tree. The 15 mRNAs were clustered into 4 groups based on the distance of pairwise correlation. **c**, GO annotation results of group 1 in biological process. **d**, GO annotation results of group 2 in biological process. **e**, GO annotation results of group 3 in biological process. **f**, GO annotation results of group 4 in biological process. The size of the dot shows the number of genes involved in the cellular biological process. The color of the dot shows the significance of the gene involved in the biological process compared with others. The Gene Ratio indicates the percentage of genes in the group involved in the biological process. One-sided Fisher’s exact test is used for the GO enrichment analysis.

All 15 ISH probe sets were hybridized overnight in fixed cells. Then, a mixture of all 15 orthogonal thermal probes were hybridized to ISH probes in situ for ~15 min. Finally, the 15 targets were imaged with iterative rounds of three-color fluorescence imaging at room temperature and heating spikes in five thermal channels (Fig. 4a). FISH images for all 15 channels are shown in Fig. 4b and an overlapped image is shown in Fig. 4c. The overall time to image 15 mRNA species in a single field of view (80 μm × 80 μm) using a high magnification objective (×100, for puncta visualization) was less than 4 min (~20 s image acquisition time for three-plex fluorescence imaging in each of the five thermal channel, ~25 s transition time (5 s heating to signal temperature, ~20 s cooling down to ~30 °C image acquisition temperature) between adjacent thermal channels) without requiring buffer exchange or fluidics. The RNA puncta clearly showed the abundance and subcellular spatial location of each mRNA target. Figure 4d shows the overlaid image for all 15 mRNAs in a single cell. The expression profile of the 15 mRNAs for eight cells is shown in Fig. 4d. The reconstructed spatial distribution and cell-to-cell covariation of the 15 mRNAs was analyzed by calculating the pairwise correlations (Extended Data Fig. 9). The genes were then clustered hierarchically into four groups based on their correlation distance between each other (Extended Data Fig. 9). We found the biological processes that the four groups of genes are involved through gene ontology analysis as shown in Extended Data Fig. 9. For example, group 4 (*ELAVAL1*, *BAZ1B*, *ARHGEF28*, *ARID1A* and *DGKD*) shows significance on the biological process including the gene regulation and chromatin assembly and disassembly.

### Thermal-plex imaging in tissues

**Fig. 5:**
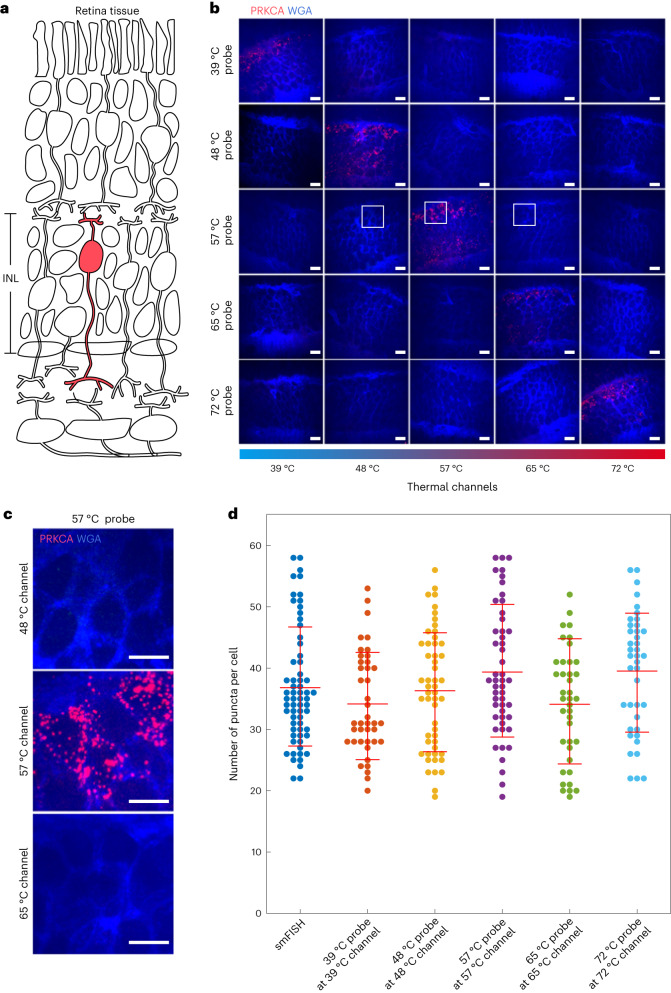
Thermal-plex imaging in retina tissue. **a**, Schematic of retina tissue and targeted cell type (RBCs, red). **b**, FISH images of the INL of the retinal tissue for five distinct thermal channels at different temperatures (39 °C, 48 °C, 57 °C, 65 °C and 72 °C) all targeting *Prkca* mRNA. All probes showed strong RNA puncta signal only in their designated temperature channel. The cell membrane was stained with WGA. **c**, Enlarged images of the retina FISH images with 57 °C thermal probes at three different temperature channels (48 °C, 57 °C and 65 °C). **d**, Plot of thermal-plex puncta for all five thermal probes in their corresponding thermal channels. smFISH puncta are used as the positive control. All five thermal channels yielded *Prkca* expression level in the INL region similar to those seen with the conventional smFISH method. The numbers of cells used to count the fluorescent puncta for smFISH and all of five thermal channels were 64, 41, 52, 50, 39 and 40, respectively. Red lines indicate the mean expression level of *Prkca* mRNA. Data are presented as mean values ± s.d. Scale bars, 10 µm. Source data

We next applied DNA thermal-plex to image RNA in fresh frozen mouse retina tissue sections. The transcriptome of the mouse retina has been reported with single-cell RNA sequencing and cell markers of bipolar cells have been validated^27^. We first designed FISH probes to target *Prkca* mRNA and validated the five thermal channels. The *Prkca* mRNA is a gene that is expressed abundantly in retinal bipolar cells located in the inner nuclear layer of the retina tissue, and plays an important role in visual processing (Fig. 5a). We added the FISH probes and then the five designed thermal probes to the formaldehyde-fixed cryosections of retina tissue (~12 µm) and performed thermal-plex imaging. As shown in Fig. 5b,c, the RNA puncta signal showed up only with the thermal probes in their designated temperature channels. No observable signal crosstalk was identified across different thermal channels. The RNA puncta were detected only in the expected region of the retina tissue. DNA thermal-plex resolved the RNA abundance at the single-cell level reliably and quantitatively for all five thermal channels similarly to the traditional smFISH method as shown in Fig. 5d.

**Fig. 6:**
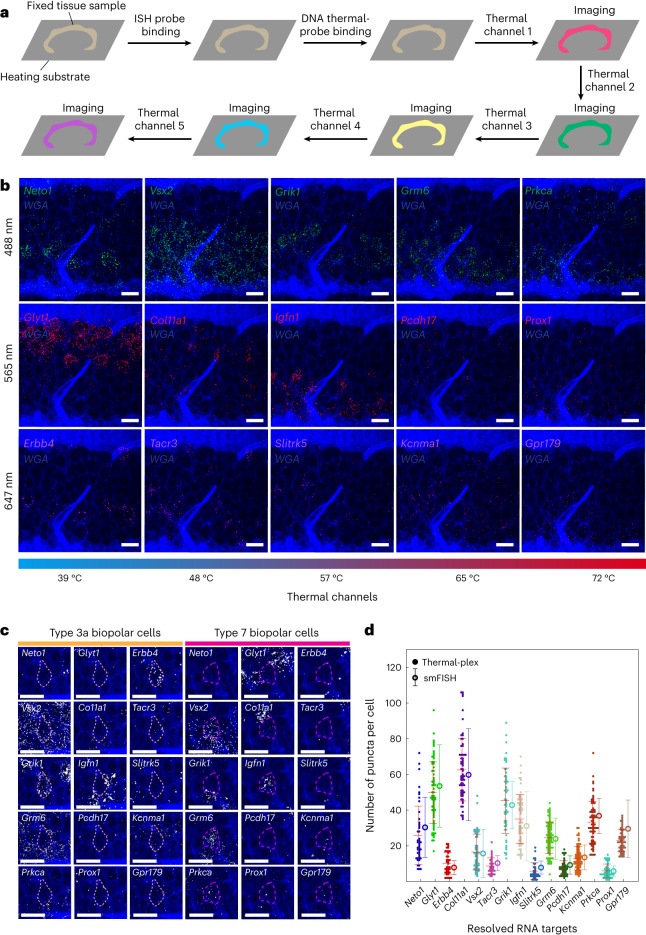
Multiplexed imaging of 15 RNA targets in retina tissue with DNA thermal-plex. **a**, Workflow of multiplexed RNA imaging with thermal-plex in retina tissue. **b**, Fluorescent images at five thermal and three fluorescent channels for the 15 RNA targets (*Neto1*, *Vsx2*, *Grik1*, *Grm6*, *Prkca*, *Glyt1*, *Col11a1*, *Igfn1*, *Pcdh17*, *Prox1*, *Erbb4*, *Tacr3*, *Slitrk5*, *Kcnma1*, *Gpr179*). Scale bars, 10 µm. **c**, Examples of classified type 3a and type 7 bipolar cells in the retinal tissue with the RNA imaged by thermal-plex. Scale bars, 10 µm. **d**, Quantitative RNA copy number per cell resolved with thermal-plex and comparisons with smFISH. Each dot represents the count in a single cell. Red horizontal lines represent the mean RNA copy numbers resolved by thermal-plex. Vertical lines indicate the statistic distributions of RNA puncta resolved by smFISH for each RNA. The numbers of cells used for quantification were *n*(*Neto1*) = 45, *n*(*Glyt1*) = 68, *n*(*Erbb4*) = 38, *n*(*Vsx2*) = 65, *n*(*Col11a1*) = 57, *n*(*Tacr3*) = 32, *n*(*Grik1*) = 58, *n*(*Igfn1*) = 69, *n*(*Slitrk5*) = 33, *n*(*Grm6*) = 85, *n*(*Pcdh17*) = 43, *n*(*Kcnma1*) = 71, *n*(*Prkca*) = 73, *n*(*Prox1*) = 48 and *n*(*Gpr179*) = 59. The number of cells was counted from at least three fields of view. The numbers of cells used for smFISH quantification were *n*(*Nego1*) = 18, *n*(*Glyt1*) = 39, *n*(*Erbb4*) = 17, *n*(*Vsx2*) = 29, *n*(*Col11a1*) = 17, *n*(*Tacr3*) = 18, *n*(*Grik1*) = 17, *n*(*Igfn1*) = 28, *n*(*Slitrk5*) = 22, *n*(*Grm6*) = 25, *n*(*Pcdh17*) = 20, *n*(*Kcnma1*) = 34, *n*(*Prkca*) = 69, *n*(*Prox1*) = 22 and *n*(*Gpr179*) = 22 (Extended Data Fig. 10). Data are presented as mean values ± s.d. Source data

**Extended Data Fig. 10:**
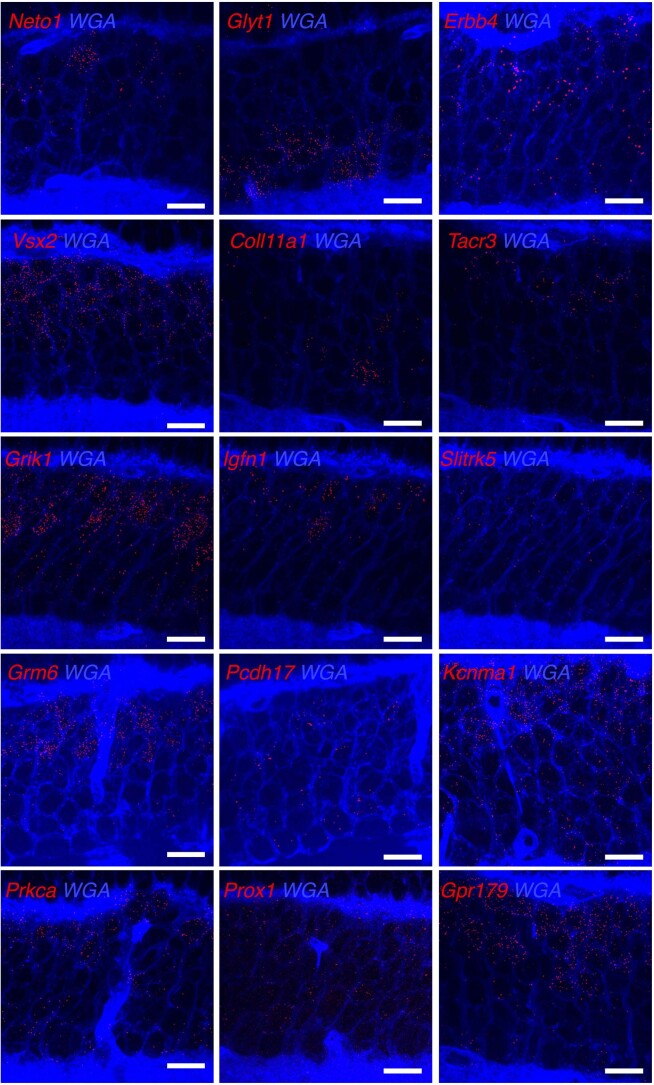
The smFISH images of 15 RNA targets in retina tissue. **a**, RNA target validation with smFISH imaging in the inner nuclear layer of retina tissue. *Neto1, Vsx2, Grik1, Grm6, and Prkca* were imaged with Atto488 dye*. Glyt1, Col11a1, Igfn1, Pcdh17, and Prox1* were imaged with Alex-565 dye. *Erbb4, Tacr3, Slitrk5, Kcnma1, and Gpr179* were imaged with Alex-647 dye. All the scale bars are 10 µm. At least three independent repeats were performed to validate the results.

Retina tissue is composed of many different types of cells. We next tested the 15 multiplexed RNA imaging by combining five thermal and three fluorescent channels. The inner nuclear layer (INL) of the retina contains several cell types, including amacrine and bipolar cells, which can be further subdivided into many functionally and transcriptionally distinct subtypes. We used DNA thermal-plex to resolve 15 different RNAs (*Neto1*, *Vsx2*, *Grik1*, *Grm6*, *Prkca*, *Glyt1*, *Col11a1*, *Igfn1*, *Pcdh17*, *Prox1*, *Erbb4*, *Tacr3*, *Slitrk5*, *Kcnma1*, *Gpr179*)^27,28^ in the INL of the retina tissue. The 15 RNA targets were assigned to temperature channels at 39 °C, 48 °C, 57 °C, 65 °C and 72 °C, respectively. We first validated thermal-plex imaging for each target individually with smFISH (Extended Data Fig. 10). We then mixed all the FISH probes to allow them to bind to the mRNA in the retina tissue simultaneously overnight (see FISH probe sequence in Supplementary Table 4). The mixtures of all DNA thermal probes were applied subsequently to bind to their assigned mRNA targets. After the application of sequential heating spikes to the five designed temperatures and imaging of the sample, all 15 RNA targets were resolved in the retina tissue with both quantitative and spatial information at single-cell level within 4 min (Fig. 6a,b). The resolved RNA targets were used to classify bipolar subcell types (Fig. 6c) according to previously reported cell RNA makers (Supplementary Table 4), such as Type 3a and Type 7 bipolar cells with markers *Erbb4* and *Igfn1*, respectively. All the resolved RNAs showed good consistency with smFISH imaging of those targets individually (Fig. 6d). All the resolved RNA targets were in the expected regions of the retina tissue (see also smFISH images in Extended Data Fig. 10). For example, *Glyt1* is expressed within a subpopulation of amacrine cells, which are located closer to the ganglion layer. *Prkca* is a marker of rod bipolar cells, which are located closer to the outer nuclear layer.

## Discussion

We describe a fluidic-free sequential multiplexed fluorescence imaging method based on iterative on-scope heating and imaging. Thermal-plex is a more rapid and accessible alternative to, or can complement, existing buffer-exchange-based sequential imaging methods. By avoiding the need for sequential incubation, washing or fluid exchange, the channel transition time is reduced from tens of minutes or hours to seconds. This transition time could potentially be reduced further with a faster heating method, for example plasmonic photothermal heating^29^. The experimental setup of thermal-plex is simple, and is compatible with standard fluorescent microscopes. The commercially available on-scope temperature control module is relatively affordable (~US$10,000), and compatible with common microscopes, although currently only offers limited fields of view. Development of even more affordable heating modules compatible with larger sample sizes would help make thermal-plex more accessible. The sample can be imaged in a fully sealed chamber to avoid buffer exchange and evaporation. As the sample is imaged in a short timeframe and on-scope, a registration step is not required in the image analysis. We expect thermal-plex to be versatile and applicable to other imaging targets beyond RNA, for example, chromosomes^19,30^ and proteins^11,31^. Although buffer-exchange-based methods can, in principle, have unlimited rounds of exchange and hence unbounded multiplexity, the multiplexity for thermal-plex is bounded by the number of thermal channels. However, incorporating cooperativity^32^ into the probe design may further narrow the thermal channel width to allow an expanded set of channels. Thermal-plex is also compatible with buffer exchange methods to further improve multiplexity. Thermal-plex is demonstrated in cells and relatively thin tissue sections. For more challenging tissue types, such as thick tissues with high background autofluorescence, signal amplification (for example, SABER^12^) could be combined with thermal-plex to improve signal-to-noise ratio and hence enable thermal-plex in more tissue types. Combining thermal-plex with combinatorial encoding^15,16^ could further enhance multiplexing level for spatially separate targets.

## Methods

### Cell culture

HeLa cells were grown in DMEM medium (Gibco, catalog no. 10569) supplemented with 10% (v/v) fetal bovine serum (Gibco, catalog no. 10082), 100 U m^−1^ penicillin, and 100 μg ml^−^^1^ streptomycin. The cells were cultured at 37 °C in the presence of 5% CO_2_. For washing and passaging, 1× DPBS (Gibco, catalog no. 14190) and 0.05% Trypsin (Gibco, catalog no. 25300) were used. HeLa (ATCC-CCL-2) and HEK293 (ATCC-CRL-1573) cell lines were used in this study.

### Design of thermal probes

The sequence of DNA thermal probes with barcode and quencher domains was first generated using NUPACK. Custom MATLAB code was used to evaluate the melting temperature of the two domains to select probes that give signal temperature at the desired thermal channels. Finally, the probability of crosstalk interactions of the selected DNA thermal probes was checked for orthogonal probe designs. All DNA thermal probe sequences are listed in Supplementary Tables 1 and 2.

### RNA FISH probe design

To design the primary ISH probes to target RNA transcripts in situ, the accession IDs of all the RNAs were first obtained, and all the sequence FASTA files were downloaded (Supplementary Table 3). The minimum probe number was 45. Then, the primary ISH probes were designed using the OligoMiner pipeline with supporting software (Jellyfish). The temperature settings were 47 °C for the lower bound and 48 °C for the higher cutoff (–*t* 47 –*T* 48) to ensure stable binding of primary probes even for the highest thermal channel. The probes were then aligned to the hg38 reference genome using bowtie2 with the -very-sensitive-local setting. Any probe that maps more than twice to the reference genome was filtered out. All primary probe sequences are listed in Supplementary Tables 3 and 4.

### DNA synthesis and probe preparation

All primary DNA probes were ordered in desalted form from Integrated DNA Technologies (IDT). The fluorophore-labeled imager strands and quencher strands were ordered with HPLC purification from IDT. Iowa black RQ was used for Atto565 and Atto488 quenching. Iowa black FQ was used for Alexa647 quenching. All primary probes for each RNA transcript were combined in an equimolar mixture at 2 μM total in RNAse- and DNAse-free ultrapure water (Invitrogen, catalog no. 10977) and stored at −20 °C until use. The quencher and imager strands were mixed in 1× PBS buffer (Invitrogen, catalog no. 00818654) at a ratio of 1.2:1 (quencher strand to imager strand) to prepare DNA thermal probes with a final probe concentration of 250 nM. The quencher strand is in excess to ensure complete quenching of imager strands.

### RNA FISH in fixed cells

HeLa cells with a concentration of 2 × 10^5^ cells per milliliter were cultured on a smart substrate (Interherence, catalog no. SmS-R-16) in an incubator (37 °C with 5% CO_2_) overnight. Culture medium was aspirated, and cells rinsed in 1× PBS buffer. Cells were then fixed in a solution containing 1× PBS buffer with 4% paraformaldehyde (v/v) (Electron Microscopy Sciences, catalog no. 191203) for 10 min, and fixation was then quenched with a solution of 100 mM NH_4_Cl for 5 min. After fixation, samples were rinsed once in 1× PBS, permeabilized in 1× PBS with 0.5% Triton X-100 (Sigma, catalog no. SLBV4122) for 10 min and washed once in 2× SSCT buffer (2× SSC buffer (Ambion, catalog no. 1501001) with 0.1% Tween-20 (Sigma, catalog no. SLCB0668)).

Primary probe sets were added to the sample in probe hybridization solution consisting of 2× SSCT, 50% formamide (v/v) (Sigma, catalog no. S4117), 10% dextran sulfate (Sigma, catalog no. 42867) and probe sets at 100 nM in total concentration. Samples were placed on a flat-block thermocycler, denatured at 60 °C for 3 min, and then incubated at 46.5 °C overnight.

After primary probe incubation, 250 μl 2× SSCT with 10% formamide (VWR, catalog no. EM-4610) which was prewarmed to 60 °C, was added to the sample, followed by aspiration. Samples were then washed with 2× SSCT with 10% formamide at 60 °C for (4 × 5 min). Finally, samples were rinsed once in 2× SSCT. For thermal probe hybridization, samples were rinsed once in 1× PBS buffer, and then 100 μl of preassembled DNA thermal probe solution with total concentration of 250 nM (with excess 50 nM of quencher strand to ensure complete quenching of the imager) in 1× PBS buffer was added. Samples were incubated at room temperature for 30 min and washed with 1× PBS buffer at 37 °C for (3 × 3 min). Samples were then stained with a solution containing in 1× PBS buffer with 4,6-diamidino-2-phenylindole (0.1 µg ml^−^^1^) for 2 min. Finally, samples were transferred to 50 μl of imaging buffer consisting of 1× PBS, 1× protocatechuic acid (Sigma, catalog no. 03930590), 1× Trolox and 1× protocatechuic dioxygenase (Sigma, catalog no. 9029-47-4).

### Retina tissue preparation

Retinas were dissected from CD-1 mice at postnatal day 18 as described previously^1^. Retinas were fixed with 4% paraformaldehyde (v/v), 0.25% Triton X (w/v) for 30 min at room temperature and then washed with PBS for 2 × 5 min. The tissue was then cryoprotected by incubation in a 7% sucrose PBS (w/v) solution for 10 min at room temperature, and then transferred to a 1:1 solution of optimal cutting temperature compound (OCT) and 30% sucrose (w/v) in PBS for 1 h. Retinas were then transferred into cryomolds and frozen in the 1:1 solution of OCT and 30% sucrose. Before tissue sectioning, eight-well ibidi chamber or heating substrate were coated with poly-d-lysine (0.3 mg ml^−^^1^ in 2× borate buffer) for 1 h at 37 °C, and then rinsed twice with water and allowed to dry completely. Embedded retinal tissue was then sectioned on a cryostat in the transverse orientation with a thickness of 14 µm. Sections within ±175 µm of the optic nerve head were collected and mounted on poly-d-lysine coated chamber slides or heating substrate. After washing with 1× PBS buffer to remove OCT, 0.5% Triton X-100 in 1× PBS buffer was used to penetrate the cells. The primary FISH probe in hybridization buffer (2× SSCT, 50% formamide (v/v), 10% dextran sulfate) was incubated with the tissue for overnight hybridization. For single-plex RNA targets, the ISH probe concentration was 100 nM. For 15-plex RNA imaging, the total probe concentration was 500 nM. The remaining procedures were the same as the cultured cell processing. For the smFISH imaging of the RNA targets, only imager of the thermal probes with a concentration of 250 nM is applied for imaging. After DNA thermal probe binding, wheat germ agglutinin (WGA) conjugated to 405s (Biotium, catalog no. 29027) was diluted to a concentration of 10 µg ml^−^^1^ in 1× PBS to stain the cell membrane of the retina tissue. Retina tissue samples were incubated for 10 min before imaging.

### Microscopy imaging

Samples were imaged on a Nikon Eclipse Ti-E microscope with Plan Apo ×100 oil-immersion objective with numerical aperture of 1.45. The microscope has a Yokogava spinning disk unit (CSU-W1, Yokogawa Electric) attached, and the excitation lasers (405 nm, 485 nm, 561 nm, 647 nm) are coupled directly into the Yokogawa W1 unit using a ×100 lens. Images were taken on an EMCCD camera (iXon3, Andor Technologies). During imaging, the temperature of the substrate was set to the desired value using the VAHEAT temperature control module (Interherence). After heating for approximately 5 s at the desired temperature (thermal channel), the temperature control module was turned off and images were taken after the temperature had cooled to below 30 °C.

Retina tissue was imaged with a Yokogawa CSU-W1 spinning disk confocal unit attached to a fully motorized Nikon Ti2 inverted microscope equipped with a Nikon linear-encoded motorized stage and an Andor Zyla 4.2 plus sCMOS camera using a Nikon Apo ×100 lens. The final digital resolution of the image was 0.16 µm per pixel.

### Theoretical simulation

Computational simulations of the thermodynamics and kinetics of DNA thermal-plex probes were performed using custom code in MATLAB. All code is available on Github (https://github.com/Albert09111/thermalplex).

### Image processing and analysis

Maximum Z projections of raw images were processed with ImageJ^2^. Image analysis including puncta analysis (identification, location and intensity information retrieval) and cell segmentation were conducted with custom MATLAB (R2020a, Mathworks) code. For puncta identification, a signal threshold was applied to the images to generate a binary image. The puncta were then identified, and the location information was retrieved. The puncta intensity was obtained as the maximum value of all the pixels the puncta covered. For cell segmentation, cell masks were created manually and then converted to a binary mask. The binary masks were applied to the original images for single-cell gene expression analysis.

To count the puncta in retina tissue sample, cells expressing the target RNAs were selected and segmented manually based on the WGA staining in 405 nm channel. For the high expression RNA targets in the target cell types (*Neto1*, *Glyt1*, *Vsx2*, *Grik1*, *Igfn1*, *Grm6*, *Prkca*, *Gpr179*), cells with no expression of the target RNA or with relatively low target RNA expression (fewer than five copies) are excluded for the statistical analysis. For other RNA targets with low expression in the target cell types, the cells with no or extremely low (fewer than two copies) expression of the RNA were excluded for the statistical analysis.

All individual fluorescent images were generated in Adobe Photoshop or ImageJ, and the brightness and contrast were adjusted linearly for display purpose. For the overlaid 15-plex mRNA images, the RNA puncta location information was retrieved from original fluorescent images and reconstructed with MATLAB scatter plot. All the custom code used for the image analysis is open source and available on Github (https://github.com/Albert09111/thermalplex).

### Hierarchical clustering analysis of the covariation in RNA and gene ontology analysis

After the abundance of 15 mRNA targets in each cell were obtained, the expression level of each was normalized to the total expression. The Pearson pairwise correlation was calculated. Then, the distance between each pair of genes was determined as 1 minus the correlation coefficient of the variation. An agglomerative hierarchical cluster tree analysis was then applied to the distance matrix to obtain the distance by using the MATLAB function linkage with average method. After the groups of genes were obtained from the hierarchical cluster analysis, the genes within each group were analyzed with gene ontology analysis^3^ by using the topGO package in R.

### Reporting summary

Further information on research design is available in the Nature Portfolio Reporting Summary linked to this article.

## Online content

Any methods, additional references, Nature Portfolio reporting summaries, source data, extended data, supplementary information, acknowledgements, peer review information; details of author contributions and competing interests; and statements of data and code availability are available at 10.1038/s41592-023-02115-3.

### Supplementary information

Supplementary Information Supplementary discussions.

Reporting Summary

Supplementary Table DNA probe sequence.

### Source data

Source Data Fig. 2 Overlapped RNA puncta count analysis and RNA puncta under different heating times.

Source Data Fig. 3 *APC* RNA counts with different thermal probes in HeLa cells.

Source Data Fig. 4 15 RNA counts in HeLa cells with thermal-plex and smFISH.

Source Data Fig. 5 *Prkca* RNA counts with different thermal probes in retina tissue.

Source Data Fig. 6 Fifteen RNA counts in retina tissue with thermal-plex and smFISH.

## Data Availability

The published article and the supplemental figures and tables include the datasets generated or analyzed during this study are available on Zenodo (10.5281/zenodo.8397563). Source data are provided with this paper.

## Code availability

The custom MATLAB code used for the binding properties of DNA thermal probes and image analysis is available at https://github.com/Albert09111/thermalplex.
