# Supplementary material for: Thermal-plex: fluidic-free, rapid sequential multiplexed imaging with DNA-encoded thermal channels
Source: Nat Methods. 2023 Dec 27;21(2):331–41. doi: 10.1038/s41592-023-02115-3 (PMC10864187; doi:10.1038/s41592-023-02115-3)
Supplement: Supplementary file 1 — Supplementary discussions. [file 41592_2023_2115_MOESM1_ESM.pdf]

nature methods

Article https://doi.org/10.1038/s41592-023-02115-3

Thermal-plex: fluidic-free, rapid sequential multiplexed imaging with DNA-encoded thermal channels

In the format provided by the
authors and unedited

Thermal-plex: Fluidic-free, rapid sequential multiplexed imaging with DNA encoded thermal channels

Fan Hong[1,2], Jocelyn Y. Kishi[1,2], Ryan N. Delgado[3], Jiyoun Jeong[1,2], Sinem K. Saka[1,2,5], Hanquan Su[1,2], Constance L. Cepko[3,4], Peng Yin[1,2*]

[1]Wyss Institute for Biologically Inspired Engineering, Harvard University, Boston, MA, USA
[2]Department of Systems Biology, Harvard Medical School, Boston, MA, USA
[3]Departments of Genetics and Ophthalmology, Blavatnik Institute, Harvard Medical School, Boston, MA, USA
[4]Howard Hughes Medical Institute, Chevy Chase, MD, USA.
[3]Present address: Genome Biology Unit, European Molecular Biology Laboratory (EMBL), Heidelberg, Germany
*Correspondence: py@hms.harvard.edu (P.Y.)

Supplementary Note 1: Three steps of DNA thermal-plex imaging.

The thermal-plex procedure consists of three steps: (1) the DNA thermal probe binding step under room temperature, (2) the probe melting step at the elevated temperature, and (3) the imaging step at room temperature. For steps 1 and 2, the system quickly reaches thermal dynamic equilibrium; for step 3, the system is metastable, with signal kinetically controlled by the slow re-association rates because of extremely low concentration of imager and quencher strands.

In the first step, DNA strands bind their targets at room temperature to encode all the signals at a high probe concentration (100 ~ 500 nM). After incubation, the excess unbound strands are washed off the sample. In this step, the equilibrium is already reached. As the melting temperature of all the DNA probe binding is much higher than the room temperature, the binding of the probes, imagers, and quenchers are in a stable binding state.

In the second step, the system is switched to a higher temperature T_n ("desired signal temperature" for probe/imager/quencher n). In this step, at the predefined thermal signal temperature T_n, the binding between the probe and the imager (for T_{n-1}), and between the quencher and the imager (for T_n) is in an unstable state, and they will all rapidly (< 1 second) dissociate with ultra-fast dissociation rate. The imager for the previous step (T_{n-1}) will rapidly dissociate to remove signal from the T_{n-1} channel, while the quencher for T_n rapidly dissociates to produce signal for the T_n channel. The equilibrium dissociated states will be reached in a few seconds. The detailed thermal dynamic analysis for the DNA thermal probes is described in Supplementary Note 2, and the kinetic analysis is described in Supplementary Note 3.

In the third step, after the melting, the temperature is switched back to the room temperature for imaging. In this step, the melted DNA strand (quenchers, imagers) concentration is extremely low and it takes prohibitively long time (tens of hours) for them to bind back to their respective targets to reach equilibrium. One round of imaging takesi much shorter time than rebinding. In this state, the system is metastable, as the actual equilibrium state is unreachable within a practically relevant time range for imaging acquisition (e.g., minutes to hours). The thermal dynamic state in the third step is about the same as the second step. Therefore, the yield of imager activation of a target is the same as in the second step. Here, we use the unbound state to approximate the equilibrium state for analysis and modeling.

Supplementary Note 2: The theoretical basis of a thermal spectrum with a DNA thermal probe.

The key mechanism of DNA thermal-plex is the generation of a fluorescent thermal spectrum by two-step melting of the DNA thermal probes *in situ*. The two melting steps are independent to each other and described as the following equations:

$$Quencher \cdot Imager \cdot Barcode \longrightarrow Quencher + Imager \cdot Barcode$$

$$Imager \cdot Barcode \longrightarrow Imager + Barcode$$

The free energy differences involved in two reactions are denoted as $\Delta G_{Quencher}$ and $\Delta G_{barcode}$, which is calculated by the following equations:

$$\Delta G_{quencher} = -(\Delta H_{quencher} - T\Delta S_{quencher})$$

$$\Delta G_{barcode} = -(\Delta H_{barcode} - T\Delta S_{barcode})$$

The enthalpic (ΔH) and entropic (ΔS) components are calculated based on the sequence of the two domains with previous measured thermodynamic parameters.[4, 5]

The fluorescent signal is modeled to be proportional to the yield of Imager-Barcode. In the first step, the fluorescent signal increases as the temperature increases. The equilibrium constant of the first step melting at a particular temperature *T* is described with the following equation:

$$\frac{[Imager \cdot Barcode]\,[Quencher]}{[Imager \cdot Barcode \cdot Quencher]} = K_{eq-quencher}$$

The yield is calculated by the following equation:

$$Yield \ = \ \frac{[Imager \cdot Barcode]}{[Quencher \cdot Imager \cdot Barcode]_0}$$

Where $[\text{Quencher-imager-barcode}]_0$ is the initial concentration of the bound DNA thermal probes.

If we assume the initial concentration of probe bound to the RNA *in situ* is a, and the concentration of imager-probe [imager-probe] = x, then we obtain the equation:

$$\frac{x^2}{a-x} = K_{eq-quencher}$$

The concentration of the signal is calculated with the quadratic formula:

$$x = \frac{-K_{eq-quencher} + \sqrt{(K_{eq-quencher})^2 - 4K_{eq-quencher}a}}{2}$$

The yield of signal is defined as φ,

$$\varphi = \frac{-K_{eq-quencher} + \sqrt{(K_{eq-quencher})^2 - 4K_{eq-quencher}a}}{2a}$$

The equilibrium constant is determined by the hybridization energy of the quencher domain:

$$K_{eq-quencher} = e^{-\frac{\Delta G_{quencher}}{RT}} = e^{\frac{\Delta H_{quencher} - T\Delta S_{quencher}}{RT}}$$

, where R is the gas constant.

Thus, the relationship between the yield and temperature for a given sequence in the quencher domain is described by the following equation:

$$\varphi = \frac{-e^{\frac{\Delta H_{quencher} - T\Delta S_{quencher}}{RT}} + \sqrt{(e^{\frac{\Delta H_{quencher} - T\Delta S_{quencher}}{RT}})^2 - 4ae^{\frac{\Delta H_{quencher} - T\Delta S_{quencher}}{RT}}}}{2a}$$

A plot of the relationship between the signal yield and the temperature is shown in Extended Data Fig. 1.

In the second step, the imager melts off from the barcode and diffuses into the buffer. This step removes the fluorescent signal *in situ* after imaging in the thermal channel and switching to higher temperature channels. The equilibrium for this second step is described by the equation bellow:

$$K_{eq-binding} = \frac{[Imager]\,[Barcode]}{[Imager \cdot Barcode]}$$

$$[Barcode] = [Imager]$$

The chemical equilibrium constant is then calculated from the hybridization energy from this region:

$$K_{eq-barcode} = e^{-\frac{\Delta G_{barcode}}{RT}}$$

The signal yield of this step is:

$$Yield = \frac{[Barcode \cdot Imager]}{[Barcode \cdot Imager]_0}$$

If we assume that in this step all quencher strands have dissociated from the primary barcode region, the initial concentration of the imager-barcode [Barcode-Imager] = a, equal to the initial concentration of the imager-quencher-barcode, and that imager-barcode concentration is y, then:

$$\frac{(a-y)^2}{y} = K_{eq-barcode}$$

By solving the equation using the quadratic formula, we get:

$$y = \frac{2a + K_{eq-barcode} + \sqrt{(2a + K_{eq-barcode})^2 - 4a^2}}{2}$$

The relationship between the signal yield θ and temperature is calculated as:

$$\theta = \frac{2a + e^{\frac{\Delta H_{barcode} - T\Delta S_{barcode}}{RT}} + \sqrt{\left(2a + e^{\frac{\Delta H_{barcode} - T\Delta S_{barcode}}{RT}}\right)^2 - 4a^2}}{2a}$$

A plot of the relationship between the signal yield and the temperature is shown in Extended Data Fig. 1b.

As the melting of two steps are independent of each other, the thermal spectrum is the overall yield combination of the two melting reaction steps.

$$\eta = \varphi\theta$$

The final thermal spectrum is shown in Extended Data Fig. 1c.

Supplementary Note 3: Kinetics of DNA imager dissociation and association in the *in situ* environment with DNA thermal-plex.

In this section, we conducted detailed kinetic analysis of DNA imagers during and after heating. Fluorescent signal is generated at a lower temperature when the quencher strand for a specific thermal channel melts off (dissociates) and is then lost again at a higher temperature when the corresponding imager strand melts off (dissociates). DNA dissociation is a fast kinetic step which can be completed in seconds at or above the melting temperature. After strands dissociate during heating steps, the temperature of the sample is cooled down to around 30 °C for imaging. However, because the strands that dissociated have diffused away in the buffer chamber and are at very low concentrations, the rate of re-binding of these strands is extremely slow.

In the quencher melting step, we can model the melting of the quencher strand during the heating steps, at a given temperature T_s, which is higher than the melting temperature of the quencher strand T_{mq}. At melting temperature of the quencher strand, the binding energy $\Delta G_{mq} = 0$. At signal temperature, the binding energy can be calculated as the following:

$$\Delta G_s = (T_s - T_{mq})\Delta S$$

The ΔS of the quencher can be calculated based on the previously well studied Santa-lucia model.7 Considering that the signal temperature is generally substantially higher than the melting temperature of the quencher, the ΔG_s of the quencher and imager strands are positive as the association is an unfavorable step. The value of ΔG_s for the quencher of thermal probes is indicated in Supplemental Table S2, ranging from 0.76 to 2.2 kcal/mol. The association rate k_{on} of the DNA strand onto the binding site is around $k_{on} = 5 \times 10^5\ M^{-1}s^{-1}$ [8], and the dissociation rate of the DNA strand at the desired signal temperature T_s can be calculated by the following equation:

$$k_{off} = k_{on} e^{\frac{\Delta G}{RT}}$$

For a thermal probe set, the differential equations of the melting steps are:

$$\frac{d\,[Quencher \cdot Imager]}{dt} = -k_{off}\,[Quencher \cdot Imager] + k_{on}\,[Quencher]\,[Imager]$$

$$\frac{d\,[Quencher]}{dt} = k_{off}\,[Quencher \cdot Imager] - k_{on}\,[Quencher]\,[Imager]$$

$$\frac{d\,[Imager]}{dt} = k_{off}\,[Quencher \cdot Imager] - k_{on}\,[Quencher]\,[Imager]$$

The strand dissociation is performed at different ΔG for different thermal probes as shown in Extended Data Fig. 1, indicating the fast strand dissociation for all the thermal probes at their signal temperatures. We then used ΔG = 1 kcal/mol for the other kinetic modeling.

For a typical mRNA, we assumed the abundance is 1~5000 copies per cell. In an imaging experiment, we usually have 10^4 cells with imaging buffer of 100 µL in the chamber. Each mRNA is designed to have 48 probes to bind. The concentration of the probe can be calculated in the range of $10^{-14} \sim 10^{-12}$ M. The cell density of a typical tissue sample, such as mouse brain, is around 10^5 cells/mm^3 on average.[9] The cell density in our experiments is at the same level of a typical brain tissue (~ 10 µm thickness). If necessary, the imaging buffer volume can be further increased to tolerate more diffused probes when imaging biological samples with even higher number of cells. To see how the RNA copy number affect the signal yield, the kinetic simulation of probe dissociation and rebind after the heating spike was performed to get the yield under different copy numbers (e.g., 1 ~ 5000 copies per cell).

By solving the equations above for a typical probe set (57 °C signal temperature) under its signal temperature (57 °C) and neighbour signal temperatures (48 °C), the dissociation curve is shown in Extended Data Fig. 1a and S3b. The dissociation of the quencher strand can be completed within 1 second. After this strand dissociates, heating is stopped, and the sample is cooled to around 30 °C for imaging. At this lower temperature, the association kinetic curve of the strand back onto the primary probe is shown in Extended Data Fig. 1c. Even after 30 hours of incubation, the rebound yield of the DNA strand is predicted to be less than 5%. Different RNA copy numbers has minimal effects on the dissociation and rebind yield of the DNA thermal probes.

Supplementary Note 4: Simulation-guided design of multiplexed DNA thermal probes

The thermal spectrum of a thermal-plex probe set is determined by the melting temperature of quencher and imager barcode region. To systematically explore the design space of DNA thermal probes, we calculated the signal yield and temperature of all the combinations of DNA barcode's melting temperatures from 40 °C to 80 °C and quencher's melting temperature from 35 ºC to 75 ºC (step size of 1°C). As the thermal spectrum of a DNA thermal probe is modeled as the multiplication of the quencher and imager strand binding curves, the melting curve of the quencher and barcode domain can be deduced from the melting temperature.

For a typical DNA melting step, the derivative of the melting curve can be fitted with a Gaussian function (shown in Extended Data Fig. 2a and 2b):

$$f(x) = \ ae^{-\frac{(x-b)^2}{2c^2}}$$

Where the resulted parameter b is the melting temperature and c is the width of the melting temperature.

We note that the higher the melting temperature, the narrower the melting curve (Extended Data Fig. 2c). To get the relationship between the melting temperature and the width of the melting curve, we randomly generated 1,000 DNA sequences with lengths from 11 nt to 40 nt and computed their melting temperatures and the width of the melting curve. At each length, we obtained the average values of the melting temperature and the width of the melting curve. The scatter plot of those generated sequences is shown in Fig. S4c. A linear fitting was applied to the scatter plot to obtain the relationship.

Based on the melting temperature of the quencher and the barcode, the yield vs temperature of the two domains can be calculated through the integration of the Gaussian function followed by min-max normalization:

$$QuencherSignal \ = \int_{20}^{T} \frac{1}{W_{quencher}\sqrt{2\pi}} e^{-\frac{\left(x-T_{m-quencher}\right)^2}{2(W_{quencher})^2}}$$

$$QuencherYield \ = \frac{QuencherSignal \ - min\,(QuencherSignal)}{max\,(QuencherSignal) - min\,(QuencherSignal)}$$

$$ImagerSignal = \int_{20}^{T} \frac{1}{W_{barcode}\sqrt{2\pi}} e^{-\frac{(x-T_{m-barcode})}{2(W_{barcode})}}$$

$$ImagerYield \ = \frac{ImagerSignal - min\,(ImagerSignal)}{max\,(ImagerSignal) - min\,(ImagerSignal)}$$

Where $W_{quencher}$ and $W_{binding}$ are the width of the quencher and barcode domain's melting curve, respectively. $T_{m-quencher}$and $T_{m-binding}$ are the melting temperatures of the quencher and barcode.

As the two melting steps are independent of each other and have reverse trends for the final fluorescent signal generation, the thermal spectrum can be calculated as:

$$SignalYield \ = \ QuencherYield \cdot (1 - ImagerYield)$$

Finally, the resultant thermal spectra for all combinations of melting temperatures of barcode regions (from 40 ºC to 80 ºC) and quencher regions (35 ºC to 75 ºC) were fitted by the Gaussian function to obtain the yield, signal temperatures, and spectrum width. Three example thermal spectra are illustrated in Extended Data Fig. 2g with different combinations of barcode and quencher domain's melting temperature. Generally,

the barcode domain has a higher melting temperature than the quencher domain to ensure relatively higher yield of fluorescent signal generation. From the set of generated spectra, we found 5 optimal combinations.

To obtain 4 channels with more stringent design speicification, we also set the signal overlap below 0.05 and ensure the yield is around 0.8; the spectra of the 4 thermal channels are shown in Extended Data Fig. i. The signal temperatures were determined to be 40 ºC, 52 ºC, 62 ºC, and 72 ºC.

Supplementary Note 5: Cost of DNA thermal-plex imaging

The cost of DNA thermal-plex RNA imaging consists of three parts: primary probe pool for RNA binding, imager of DNA thermal probes, and quencher of DNA thermal probe. A typical probe pool of ~50 unpurified probe oligos costs around ~$300, which would give enough quantities for 250 mL 1 μM of probe pool. A typical imager costs $200 for ~10 nmole yield, giving 1 mL of 1 μM solution. A typical quencher costs $80 for ~15 nmole yield, giving 1.5 mL of 1 μM solution.

In a typical thermal-plex RNA imaging experiment, ~120 μL of ISH probe with concentration of 100 nM is used, which costs around $0.012. The usage of DNA thermal probe is usually 120 μL of 200 nM, which costs around $8 per experiment.

The cost could be further reduced if unlabeled DNA oligo is ordered, and the fluorophore and quencher conjugation is performed in-house.

References:

1. West, E.R. et al. Spatiotemporal patterns of neuronal subtype genesis suggest hierarchical development of retinal diversity. *Cell Rep* **38**, 110191 (2022).
2. Schindelin, J. et al. Fiji: an open-source platform for biological-image analysis. *Nat Methods* **9**, 676-682 (2012).
3. Ashburner, M. et al. Gene ontology: tool for the unification of biology. The Gene Ontology Consortium. *Nat Genet* **25**, 25-29 (2000).
4. SantaLucia, J., Jr., Allawi, H.T. & Seneviratne, P.A. Improved nearest-neighbor parameters for predicting DNA duplex stability. *Biochemistry* **35**, 3555-3562 (1996).
5. Allawi, H.T. & SantaLucia, J., Jr. Thermodynamics and NMR of internal G.T mismatches in DNA. *Biochemistry* **36**, 10581-10594 (1997).
6. Beliveau, B.J. et al. OligoMiner provides a rapid, flexible environment for the design of genome-scale oligonucleotide in situ hybridization probes. *Proc Natl Acad Sci U S A* **115**, E2183-E2192 (2018).
7. SantaLucia, J., Jr. & Hicks, D. The thermodynamics of DNA structural motifs. *Annu Rev Biophys Biomol Struct* **33**, 415-440 (2004).
8. Ostromohov, N., Huber, D., Bercovici, M. & Kaigala, G.V. Real-Time Monitoring of Fluorescence in Situ Hybridization Kinetics. *Anal Chem* **90**, 11470-11477 (2018).
9. Keller, D., Ero, C. & Markram, H. Cell Densities in the Mouse Brain: A Systematic Review. *Front Neuroanat* **12**, 83 (2018).
