# Supplementary material for: Thermal-plex: fluidic-free, rapid sequential multiplexed imaging with DNA-encoded thermal channels
Source: Nat Methods. 2023 Dec 27;21(2):331–41. doi: 10.1038/s41592-023-02115-3 (PMC10864187; doi:10.1038/s41592-023-02115-3)
Supplement: Supplementary file 2 — Reporting Summary [file 41592_2023_2115_MOESM2_ESM.pdf]

nature portfolio

Corresponding author(s): Peng Yin

Last updated by author(s): 2023/03/27

Reporting Summary

Nature Portfolio wishes to improve the reproducibility of the work that we publish. This form provides structure for consistency and transparency in reporting. For further information on Nature Portfolio policies, see our Editorial Policies and the Editorial Policy Checklist.

Statistics

For all statistical analyses, confirm that the following items are present in the figure legend, table legend, main text, or Methods section.

n/a	Confirmed	
☐	☒	The exact sample size (*n*) for each experimental group/condition, given as a discrete number and unit of measurement
☐	☒	A statement on whether measurements were taken from distinct samples or whether the same sample was measured repeatedly
☐	☒	The statistical test(s) used AND whether they are one- or two-sided<br>*Only common tests should be described solely by name; describe more complex techniques in the Methods section.*
☒	☐	A description of all covariates tested
☐	☒	A description of any assumptions or corrections, such as tests of normality and adjustment for multiple comparisons
☐	☒	A full description of the statistical parameters including central tendency (e.g. means) or other basic estimates (e.g. regression coefficient) AND variation (e.g. standard deviation) or associated estimates of uncertainty (e.g. confidence intervals)
☐	☒	For null hypothesis testing, the test statistic (e.g. *F*, *t*, *r*) with confidence intervals, effect sizes, degrees of freedom and *P* value noted<br>*Give P values as exact values whenever suitable.*
☒	☐	For Bayesian analysis, information on the choice of priors and Markov chain Monte Carlo settings
☒	☐	For hierarchical and complex designs, identification of the appropriate level for tests and full reporting of outcomes
☐	☒	Estimates of effect sizes (e.g. Cohen's *d*, Pearson's *r*), indicating how they were calculated

Our web collection on statistics for biologists contains articles on many of the points above.

Software and code

Policy information about availability of computer code

Data collection	Micromanager and Nikon Element were used to acquire images.
Data analysis	ImageJ2/FIJI for Z max projection, Adobe photoshop (version 2023) for image processing.<br>NUPACK3.2.0 was used to calculate cross-hybridization probabilities.<br>OligoMiner, Bowtie2, Jellyfish, BLASTn were used to design and process the probe sequences. Puncta were identified and assigned to single cells using custom MATLAB (version 2020) pipeline (https://github.com/Albert09111/thermalplex).

For manuscripts utilizing custom algorithms or software that are central to the research but not yet described in published literature, software must be made available to editors and reviewers. We strongly encourage code deposition in a community repository (e.g. GitHub). See the Nature Portfolio guidelines for submitting code & software for further information.

Data

Policy information about availability of data

All manuscripts must include a data availability statement. This statement should provide the following information, where applicable:

- Accession codes, unique identifiers, or web links for publicly available datasets
- A description of any restrictions on data availability
- For clinical datasets or third party data, please ensure that the statement adheres to our policy

Provide your data availability statement here.

Human research participants

Policy information about studies involving human research participants and Sex and Gender in Research.

Reporting on sex and gender	N/A
Population characteristics	N/A
Recruitment	N/A
Ethics oversight	*Identify the organization(s) that approved the study protocol.*

Note that full information on the approval of the study protocol must also be provided in the manuscript.

Field-specific reporting

Please select the one below that is the best fit for your research. If you are not sure, read the appropriate sections before making your selection.

☒ Life sciences ☐ Behavioural & social sciences ☐ Ecological, evolutionary & environmental sciences

For a reference copy of the document with all sections, see nature.com/documents/nr-reporting-summary-flat.pdf

Life sciences study design

All studies must disclose on these points even when the disclosure is negative.

Sample size	For the puncta quantification, the sample size was determined by the number of positive cells and puncta within the replicates. n=3 was chosen as minimal. We determined this to be sufficient because of internal control and low observed variability
Data exclusions	Data were not excluded from analysis
Replication	All replication attempts were successful and observed marker expression patterns with orthogonal methods and previously known results. All the experiments have been repeated independently at least three times across by at least three different people in the past 2 years.
Randomization	All cells and puncta that passed quality control were analyzed equally with no sub-sampling and thus, there were no requirement for randomization
Blinding	Blinding was not possible as experimental conditions were evident from the image data. Quantification were performed using computational pipeline applied equally to all conditions that replicates for a given probe. Thresholds were applied for each probe on images.

Reporting for specific materials, systems and methods

We require information from authors about some types of materials, experimental systems and methods used in many studies. Here, indicate whether each material, system or method listed is relevant to your study. If you are not sure if a list item applies to your research, read the appropriate section before selecting a response.

Materials & experimental systems

n/a	Involved in the study	
☒	☐	Antibodies
☐	☒	Eukaryotic cell lines
☒	☐	Palaeontology and archaeology
☐	☒	Animals and other organisms
☒	☐	Clinical data
☒	☐	Dual use research of concern

Methods

n/a	Involved in the study	
☒	☐	ChIP-seq
☒	☐	Flow cytometry
☒	☐	MRI-based neuroimaging

Eukaryotic cell lines

Policy information about cell lines and Sex and Gender in Research

Cell line source(s)	Hela Cells (ATCC), and HEK293 cell line (ATCC)
Authentication	None of the cell lines have been authenticated
Mycoplasma contamination	Cell lines were not tested for mycoplasma contamination but no indication of contamination was observed.
Commonly misidentified lines (See ICLAC register)	No commonly misidentified cell lines were used in this study.

Animals and other research organisms

Policy information about studies involving animals; ARRIVE guidelines recommended for reporting animal research, and Sex and Gender in Research

Laboratory animals	C57BL/6NCrl mice at postnatal day 18. Mice were housed in a barrier facility with a 12-h light/12-h dark cycle and temperature and humidity control (70 °F, 50% rack humidity)
Wild animals	No wild animals were used in this study.
Reporting on sex	no sex is asigned
Field-collected samples	No field-collected samples were used in this study.
Ethics oversight	The mouse work was performed under the study protocol IS00001679, as approved by the Institutional Animal Care and Use Committee.

Note that full information on the approval of the study protocol must also be provided in the manuscript.
